# Disabled people’s experiences accessing healthcare services during the COVID-19 pandemic: a scoping review

**DOI:** 10.1186/s12913-023-09336-4

**Published:** 2023-04-06

**Authors:** Karen McBride-Henry, Solmaz Nazari Orakani, Gretchen Good, Michael Roguski, Tara N. Officer

**Affiliations:** 1 School of Nursing, Midwifery, and Health Practice, Wellington Faculty of Health, Victoria University of Wellington, Wellington, New Zealand; 2 School of Health Sciences, Massey University, Palmerston North, New Zealand; 3 Kaitiaki Research and Evaluation, Wellington, New Zealand

**Keywords:** Disability, Healthcare, Healthcare access, COVID-19, Qualitative methods, Consumer voice, Experiences, Scoping review

## Abstract

**Background:**

Disruptions to healthcare services during the COVID-19 pandemic are well-recognised problems. However, a dearth of research exists on disabled people’s experiences with accessing these services. A scoping review was undertaken to identify and explore research on the experience of disabled people in accessing healthcare services between 2020 and 6 February 2023.

**Methods:**

PubMed, Web of Science, CINAHL, and OVID were employed to search for research that included the voice of disabled people, or their parents or caregivers. Over two distinct phases, a total of 2,201 articles were reviewed according to article titles, keywords, and abstracts. Eighty-one studies were identified that met the inclusion criteria; these were reviewed in full.

**Results:**

Eighteen studies specifically described the experiences of accessing healthcare or disability services, and sixty-three raised healthcare challenges as a secondary consideration. Many disabled people struggled to access healthcare services and felt they were invisible; as a result, individuals’ mental health was negatively affected. Disabled people with compounding vulnerabilities were at the most risk of experiencing a lack of healthcare access.

**Conclusions:**

There is an urgent need for research and policy that is responsive to disabled people’s access to healthcare during the pandemic; currently many health policies are ‘disability-blind’ and exclude these members of the global community. Furthermore, to assist in creating disability-responsive research, funding needs to prioritise researchers within the disabled community.

## Background

Disabled people constitute approximately 15% of the world’s population [1, 2]; the World Health Organization [2] estimates that the number of disabled people is growing rapidly, in part because of population ageing and the impacts of long-COVID [3]. Disabled people access healthcare services at approximately double the rate of those who are not disabled [4, 5], and are more likely to have concurrent chronic health illnesses [2, 6–8]. In addition, when disabled people access healthcare, they likely encounter discrimination and other barriers, including obstacles, attitudes and actions that impact the quality of health services [2, 5, 9, 10]. Furthermore, disruptions to these services disproportionately impact this population through treatment delays and associated mental distress [11–16] and poorer health outcomes over time [2, 12, 13, 17–19]. Despite comprising a significant proportion of the population, disabled people are especially vulnerable to precarious healthcare access resulting from ableist structures.

While barriers to disabled people’s healthcare access have been long appreciated, the COVID-19 pandemic created unprecedented global health system challenges [16, 20–22]. Such challenges include significant disruption to healthcare delivery and provision of timely services [2, 14, 16, 20–34]. For example, access to rehabilitation and occupational therapy support services was significantly impacted [24, 25]. Disabled people also had reduced or no access to health and disability services, likely negatively impacting this population’s long-term health [24, 31, 35, 36].

A growing body of evidence emphasises the importance of knowing disabled people’s lived realities [30, 37–39]. This can be especially appreciated given disabled people’s experiences are often ignored because of the privileged position of ableism [13, 40]. Significantly, disabled people’s continued marginalisation has been perpetuated through the imposition of ableist service designs and lack of external responsiveness to disabled people’s lived realities, needs and direction [2, 37]. Further, aligned with ableist privileging, Brennan [37] cautions that the epistemological positioning of those who conduct research on disabled people, and the development of disability-related policy, needs to be critically evaluated. Such caution is founded on a wariness that research on marginalised populations, without their endorsement, involvement or critical appraisal can result in the marginalised population’s continued misrepresentation and, as such, failure to address healthcare access needs.

Health systems geared to cater to disabled people are better designed and deliver services more effectively when people with lived experiences provide input [2, 30, 37, 41]. This is because unique insights from those accessing healthcare enable service delivery to be specifically tailored to meet people’s needs, take into account their unique strengths, and identify areas where additional supports are required [41, 42]. The COVID-19 pandemic exacerbated challenges in accessing healthcare for disabled people [13, 32, 37, 43]. Within this context, there is a need to understand the implications of this loss of access on disabled communities. The present scoping review explores what COVID-19 pandemic research includes disabled people, or primary caregivers’, voices about the experience of accessing healthcare during 2020–2023.

## Methods

Our review reflected Arksey and O’Malley’s methodology for scoping reviews and the PRISMA extension for scoping reviews [44–46]. We applied strict criteria (Table 1) to our search of PubMed, Web of Science, CINAHL, and OVID (including MEDLINE). The search occurred in two distinct phases: phase one occurred on the week of 18 April 2022, and phase two, the week of 6 February 2023. Phase one included articles published after 1 January 2020 and focussed on disabled people accessing healthcare during the COVID-19 pandemic. Phase two aligned with phase one and included articles published between the initial search to 6 February 2023. Search terms (and relevant variations, see Table 2) included “COVID-19”, “health*”, “access” “disabled people” and “disability”. The search strategy, in keeping with Arksey and O’Malley’s methodology [45], was purposely broad, and an in-depth analysis of articles was conducted to ensure that the research team captured a greater breadth of findings.

**Table 1:** Inclusion and exclusion criteria

**Inclusion criteria**
• Presented the voice/ experiences of disabled people or their parents/guardians around accessing health services during COVID-19
• Involved qualitative designs, self-reported accounts, or open-ended survey responses
• Covered privately and publicly funded community, primary, secondary, tertiary, acute care, or hospital healthcare settings
**Exclusion criteria**
• Non-English language publications
• Published before 01/01/2020
• Treatment or intervention studies and clinical trials
• Non-research articles, editorials, opinion pieces, conference abstracts and proceedings
• Epidemiological papers and research protocols
• Healthcare services provided by non-registered health professionals
• Animal studies

**Table 2:** Search term variations and filters

Disability	Disabil* OR disabl* OR “disabled persons” OR “disabled children” OR “persons with disabilities” OR impair* OR special OR “special needs”
Health	health* OR “health care” OR “health services” OR “healthcare access”
COVID-19	covid-19 OR coronavirus OR 2019-ncov OR sars-cov-2 OR cov-19
Filters	Published during 2020–2022
	English
	Peer-reviewed research

All articles were screened against the inclusion/ exclusion criteria using the article title, keywords and abstract; if there were concerns about suitability for inclusion full texts were also reviewed, in keeping with the methodological approach. In phase one, GG, KMH, SNO, and TNO screened all articles. The team used Rayyan (Rayyan Systems Inc, 2023) to support the review including when identifying duplicates and reviewing conflicts in screened articles. Any phase one conflicts that were unable to be resolved were reviewed by MR. In phase two, all articles were screened by KMH and TNO, who then discussed conflicts, MR was available to review any conflicts that were unable to be resolved. For an article to be included in the review it needed to have addressed healthcare access, which often required a thorough analysis of the entire paper; this review of papers occurred during the conflict resolution phase. All included full-text articles in both study phases were then reviewed by TNO and KMH using the PRISMA Statement 2020 checklist as an evaluation tool [44].

### Data extraction and analysis

Data were extracted by TNO and KMH into a Microsoft Excel (Microsoft Corporation, 2023) spreadsheet after the research team agreed on a refined evaluation criterion; these were as follows: study title, authors, research and study design, study focus, objectives, participant description and outcomes. A further sub-analysis explored primary author characteristics; this sub-analysis included fields such as apparent gender, funding source, the researchers’ discipline, explicit disability community connections, and if research team members were part of the disabled community (Table 3). Some of this analysis was challenged by reporting differences.

**Table 3:** Author characteristics and project funding status

Author	Does research focus solely on disabled populations?	Funding awarded for study	Primary author’s gender	Academic position of the primary author at publication	Explicit inclusion of disabled researchers in the research team?	Explicit engagement with the disabled community?
**Phase one**						
Bailey, A., Harris, MA., Bogle, D., Jama, A., Muir, SA., Miller, S., Walters, CA., & Govia I	No	Yes	Female	Lecturer	None stated	No
Dai, R., & Hu L	Yes	Not stated	Male	Not found	Yes	Yes
Embregts, P., Heerkens, L., Frielink, N., Giesbers, S., Vromans, L., & Jahoda, A	Mothers of disabled children	Not stated	Female	Professor	None stated	Yes
Epstein, S., Campanile, J., Cerilli, C., Gajwani, P., Varadaraj, V., & Swenor, BK	Yes	Yes	Female	Student	Yes	Yes
Kim, MA., Yi, J., Sung, J., Hwang, S., Howey, W. & Jung, SM	Yes	None	Female	Associate Professor	Yes	Yes
Kwegyir Tsiboe, A	Yes	Not stated	Male	PhD Candidate	None stated	Not stated
Lindsay, S., Ahmed, H., & Apostolopoulos, D	No	Yes	Female	Senior Scientist	None stated	Yes
Mathias, K., Rawat, M., Philip, S., & Grills, N	No	Partial	Female	Senior Lecturer	None stated	Not stated
Reber, L., Kreschner, JM., DeShong, GL., & Meade, MA	Yes	Yes	Female	Post-Doctoral Fellow	Yes	Yes
Saunders, GH., Jackson IR., & Visram, AS	No	Yes	Female	Senior Research Fellow	None stated	Not stated
Schwartz, AE., Munsell, EGS., Schmidt, EK., Colón-Semenza, C., Carolan, K., & Gassner, DL	No	Not stated	Female	Assistant Professor	Yes	Yes
Sutter, EN., Smith Francis, L., Francis, SM., Lench, DH., Nemanich, ST., Krach, LE., Sukal-Moulton, T. & Gillick, BT	Parents of disabled children	Yes	Female	PhD Candidate	None stated	Not stated
Theis, N., Campbell, N., De Leeuw, J., Owen, M., & Schenke, KC	Parents of disabled children	Not stated	Female	Senior Lecturer	None stated	Yes
Xu, D., Yan, C., Zhao, Z., Weng, J., & Ma, S	Yes	Yes	Female	Associate Professor	Yes	Yes
**Phase two**						
AlMeraj, Z., Abu Doush, I., Alhuwail, D., Shama, S., AlBahar, A., & Al-Ramahi, M	Yes	Not stated	Female	Assistant Professor	None stated	Yes
Arbour-Nicitopoulos, KP., James, ME., Moore, SA., Sharma, R., & Martin Ginis, KA	Yes	Yes	Female	Associate Professor	None stated	None stated
Arichi, T., Cadwgan, J., McDonald, A., Patel, A., Turner, S., Barkey, S., Lumsden, DE., & Fairhurst, C	Yes	Not stated	Male	Clinical Senior Lecturer	None stated	No
Bellon, M., Idle, J., Lay, K., & Robinson, S	Yes	Yes	Female	Associate Professor	None stated	Yes
Bergmans, RS., Chambers-Peeple, K., Aboul-Hassan, D., Dell’Imperio, S., Martin, A., Wegryn-Jones, R., Xiao, LZ., Yu, C., Williams, DA., Clauw, DJ., & DeJonckheere, M	Yes	Yes	Female	Research Investigator	None stated	No
Binder-Olibrowska, KW., Wrzesińska, MA., & Godycki-Ćwirko, M	Yes	Yes	Female	PhD Candidate	None stated	No
Bozkus-Genc, G., & Sani-Bozkurt, S	Yes	Not stated	Female	Assistant Professor	None stated	No
Burke, MM., Cheung, WC., Li, C., DaWalt, L., Segal, J., & Taylor, JL	Yes	Yes	Female	Professor	None stated	No
Buse, DC., Gerstein, MT., Houts, CR., McGinley, JS., Uzumcu, AA., McCarrier, KP., Cooke, A., Touba, NM., Nishida, TK., Wirth, RJ., & Lipton, RB	Yes	Yes	Female	Professor	None stated	Yes
Caldwell, J., Heyman, M., Atkins, M., & Ho, S	Yes	Yes	Male	Senior Scientist	None stated	Yes
Chaiban, L., Benyaich, A., Yaacoub, S., Rawi, H., Truppa, C., & Bardus, M	Yes	Not stated	Female	Research Assistant	None stated	No
Chirico, I., Ottoboni, G., Giebel, C., Pappadà, A., Valente, M., Degli Esposti, V., Gabbay, M., & Chattat, R	Yes	Yes	Female	Post-Doctorate Fellow	None stated	For recruitment purposes only
Chowdhury, S., Urme, SA., Nyehn, BA., Mark, HR., Hassan, MT., Rashid, SF., Harris, NB., & Dean, L	Yes	Yes	Female	Research Assistant	Yes	Yes
Cochran, AL., McDonald, NC., Prunkl, L., Vinella-Brusher, E., Wang, J., Oluyede, L., & Wolfe, M	No	Yes	Female	Assistant Professor	None stated	None stated
Costa, B., McWilliams, D., Blighe, S., Hudson, N., Hotton, M., Swan, MC., & Stock, NM	No	Not stated	Female	Research Fellow	None stated	No
Currie, G., Finlay, B., Seth, A., Roth, C., Elsabbagh, M., Hudon, A., Hunt, M., Jodoin, S., Lach, L., Lencucha, R., Nicholas, DB., Shakako, K., & Zwicker, J	Yes	Yes	Female	Associate Professor	None stated	Yes
Dean, NA., Marwaha, A., Grasdal, M., Leong, S., Mesa, A., Krassioukov, AV., & Bundon, A	Yes	Yes	Male	PhD Candidate	Not stated	None stated
Dodds, RL., Maurer, KJ., Montgomery, LS., Cutting, S., & Jilek, C	Yes	Not stated	Female	PhD Candidate	Yes, parent advocate	None stated
Filbay, S., Bennell, K. L., Morello, R., Smith, L., Hinman, R. S., & Lawford, BJ	Yes	Yes	Female	Senior Research Fellow	None stated	No
Filler, T., Benipal, P. K., Minhas, R. S., & Suleman, S	Yes	Yes	Female	MD Candidate	None stated	No
Forslund, T., Fernqvist, S., & Tegler, H	Yes	Yes	Male	Post-Doctorate Fellow	None stated	No
Fridell, A., Norrman, H. N., Girke, L., & Bölte, S	Yes	Not stated	Female	PhD Candidate	None stated	No
Goddard, K. S., Schulz, J., Nzuki, I., & Hall, J. P	Yes	Yes	Female	Associate Researcher	None stated	Yes
Good, G., Nazari Orakani, S., Officer, T., Roguski, M., & McBride-Henry, K	Yes	Yes	Female	Senior Lecturer	Yes	Yes
Goodley, D., Lawthom, R., Liddiard, K., & Runswick-Cole, K	Yes	Yes	Male	Professor	None stated	Yes
Govia, I., Palmer, T., Stubbs, M., Harris, M., Bogle, D., Miller, S., Walters, C., Muir, S. A., & Bailey, A	No	Not stated	Female	Senior Lecturer	None stated	No
Gul, S., & Ygmur, Y	Yes	Not stated	Female	Assistant Professor	None stated	No
Hall, K. A. E., Deusdad, B., D’Hers Del Pozo, M., & Martínez-Hernáez, Á	Yes	Yes	Female	PhD Candidate	Yes	Yes
Hielscher, L., Ludlow, A., Mengoni, S. E., Rogers, S., & Irvine, K	Yes	Yes	Female	PhD Candidate	None stated	Yes
Hochman, Y., Shpigelman, C.-N., Holler, R., & Werner, S	Yes	Not stated	Female	Senior Lecturer	Yes	Yes
Isensee, C., Schmid, B., Marschik, P. B., Zhang, D., & Poustka, L	Yes	Not stated	Female	Psychologist	None stated	No
LaVela, S. L., Wu, J., Nevedal, A. L., Harris, A. H. S., Frayne, S. M., Arnow, K. D., Barreto, N. B., Davis, K., & Eisenberg, D	Yes	Not stated	Female	Research Associate Professor	None stated	Yes
Linden, M. A., Forbes, T., Brown, M., Marsh, L., Truesdale, M., McCann, E., Todd, S., & Hughes, N	Yes	Yes	Male	Reader	None stated	No
Mazzoni, N., Bentenuto, A., Filosofi, F., Tardivo, A., Strathearn, L., Zarei, K., De Falco, S., Venuti, P., Iandolo, G., & Giannotti, M	Yes	Not stated	Female	Teaching Fellow	None stated	No
Mbazzi, F. B., Nalugya, R., Kawesa, E., Nimusiima, C., King, R., van Hove, G., & Seeley, J	Yes	Yes	Female	Assistant Professor	None stated	Yes
Mitwalli, S., Kiwan, D., Abdul-Samad, L., & Giacaman, R	Yes	Yes	Female	Academic researcher, otherwise unspecified	None stated	No
Mohamed, H., Wamera, E., & Malima, W	Yes	Yes	Male	Academic researcher, otherwise unspecified	None stated	No
Navas, P., Verdugo, M. Á., Martínez, S., Amor, A. M., Crespo, M., & Deliu, M. M	Yes	Yes	Female	Associate Professor	None stated	No
Nguyen, L., & Bui, M	No	Yes	Female	Senior Lecturer	None stated	No
Nicholas, D. B., Zulla, R. T., Conlon, O., Dimitropoulos, G., Urschel, S., Rapoport, A., Katz, S. L., Bruce, A., West, L. J., Belletrutti, M., Cullen, E., & Zwaigenbaum, L	Yes	Yes	Male	Professor	None stated	No
Oude Lansink, I. L. B., van Stam, P. C. C., Schafrat, E. C. W. M., Mocking, M., Prins, S. D., Beelen, A., Cuppen, I., van der Pol, W. L., Gorter, J. W., & Ketelaar, M	Yes	Not stated	Female	Physiatrist	None stated	No
Pellicano, E., Brett, S., den Houting, J., Heyworth, M., Magiati, I., Steward, R., Urbanowicz, A., & Stears, M	Yes	Yes	Female	Professor	Yes	Yes
Pincock, K., Jones, N., Baniodeh, K., Iyasu, A., Workneh, F., & Yadete, W	Yes	Not stated	Female	Research Officer	None stated	No
Pinkerton, L. M., Murphy, A., Bruckner, E., & Risser, H	Yes	Not stated	Female	PhD Candidate	Yes advocate	Yes
Portillo-Aceituno, A., Calderón-Bernal, A., Pérez-Corrales, J., Fernández-de-Las-Peñas, C., Palacios-Ceña, D., & Güeita-Rodríguez, J	Yes	Not stated	Female	Paediatric physiotherapist	None stated	No
Roguski, M., Officer, T., Nazari Orakani, S., Good, G., Händler-Schuster, D., & McBride-Henry, K	Yes	Yes	Male	Research Director	Yes	Yes
Rohn, E. J., Hearn, J. H., Philippus, A. M., & Monden, K. R	Yes	Not stated	Male	Assistant Professor	None stated	No
Sage, R., Standley, K., & Ipsen, C	Yes	Yes	Female	Project Director	None stated	No
Saketkoo, L. A., Jensen, K., Nikoletou, D., Newton, J. J., Rivera, F. J., Howie, M., Reese, R. K., Goodman, M., Hart, P. B., Bembry, W., Russell, A., Lian, I., Lammi, M. R., Scholand, M. B., & Russell, A.-M	Yes	Yes	Female	MD, Associate Professor	Yes	Yes
Sarica, A. D., Ulu-Ercan, E., & Coşkun, U. H	Yes	Not stated	Female	Associate Professor	None stated	No
Sarker, D., Shrestha, S., & Tamang, S. K. B	Yes	Not stated	Male	Academic researcher, otherwise unspecified	None stated	No
Scherer, N., Wiseman, P., Watson, N., Brunner, R., Cullingworth, J., Hameed, S., Pearson, C., & Shakespeare, T	Yes	Yes	Male	Research Fellow	None stated	Yes
Sebring, J. C. H., Capurro, G., Kelly, C., Jardine, C. G., Tustin, J., & Driedger, S. M	Yes	Yes	Non-binary	PhD candidate	Yes	Yes
Selick, A., Bobbette, N., Lunsky, Y., Hamdani, Y., Rayner, J., & Durbin, J	Yes	Yes	Female	Project Scientist	None stated	Yes
Sellmaier, C., & Kim, J	Yes	Not stated	Female	Assistant Professor	None stated	No
Sharma, Y., Whiting, A., & Dutta, T	Yes	Yes	Female	PhD Candidate	None stated	No
Silver, H., Rosselot, H., Shaffer, R., & Lozano, R	Yes	Yes	Female	Not found	None stated	No
Smythe, T., Mabhena, T., Murahwi, S., Kujinga, T., Kuper, H., & Rusakaniko, S	Yes	Yes	Female	Associate Professor	None stated	No
Solomon Sanders, J., Rajapillai L. I. Pillai, R., Sturley, R., Sillau, S., Asato, M., R., B., Aravamuthan, B., Bonuck, K., Cervenka, M., Hammond, N., Siegel, J., Siasoco, V., & Margolis, B	Yes	Not stated	Female	MD, Assistant Professor	None stated	No
Tetali, S., Kamalakannan, S., Sadanand, S., Lewis, M. G., Varughese, S., Hans, A., & Murthy, G. V. S	Yes	Yes	Female	Associate Professor	None stated	No
Toccalino, D., Haag, H. L., Estrella, M. J., Cowle, S., Fuselli, P., Ellis, M. J., Gargaro, J., & Colantonio, A	Yes	Not stated	Female	PhD Candidate	None stated	Yes
Turcheti, N., Laurent, A. A., Delgado, C., Sainati, K., Johnson, K., & Wong, E. Y	Yes	Yes	Female	Social Research Scientist	None stated	Yes
Vestal, LE., Schmidt, AM., Dougherty, NL., Sherby, MR., Newland, JG., & Mueller, NB. for the COMPASS-T Study Group	Yes	Not stated	Female	Evaluation Manager	None stated	Yes
Waltz, M., Canter, C., Bensen, JT., Berg, JS., Foreman, AK. M., Grant, TL., Hassmiller Lich, K., Navas, A., O’Daniel, JM., Powell, BC., Rini, CM., Staley, BS., & Cadigan, RJ	Yes	Yes	Female	Research Associate	None stated	No
Wanjagua, R., Hepburn, S.-J., Faragher, R., John, S. T., Gayathri, K., Gitonga, M., Meshy, C. F., Miranda, L., & Sindano, D	Yes	Not stated	Female	PhD Candidate	Yes	Yes
Xu, D., Ma, S., Yan, C., & Zhao, Z	Yes	Yes	Female	Assistant Professor	None stated, but fluent in Chinese Sign Language	Yes
Zebehazy, KT., Rosenblum, LP., & Thompson, KM	Yes	Not stated	Female	Professor	None stated	No

KMH and TNO examined the final extracted studies from both research phases and analysed these thematically following scoping review aims. These themes were then systematically explored and shared with the wider research team for verification and refinement.

## Results

### Overview

**Fig. 1:**
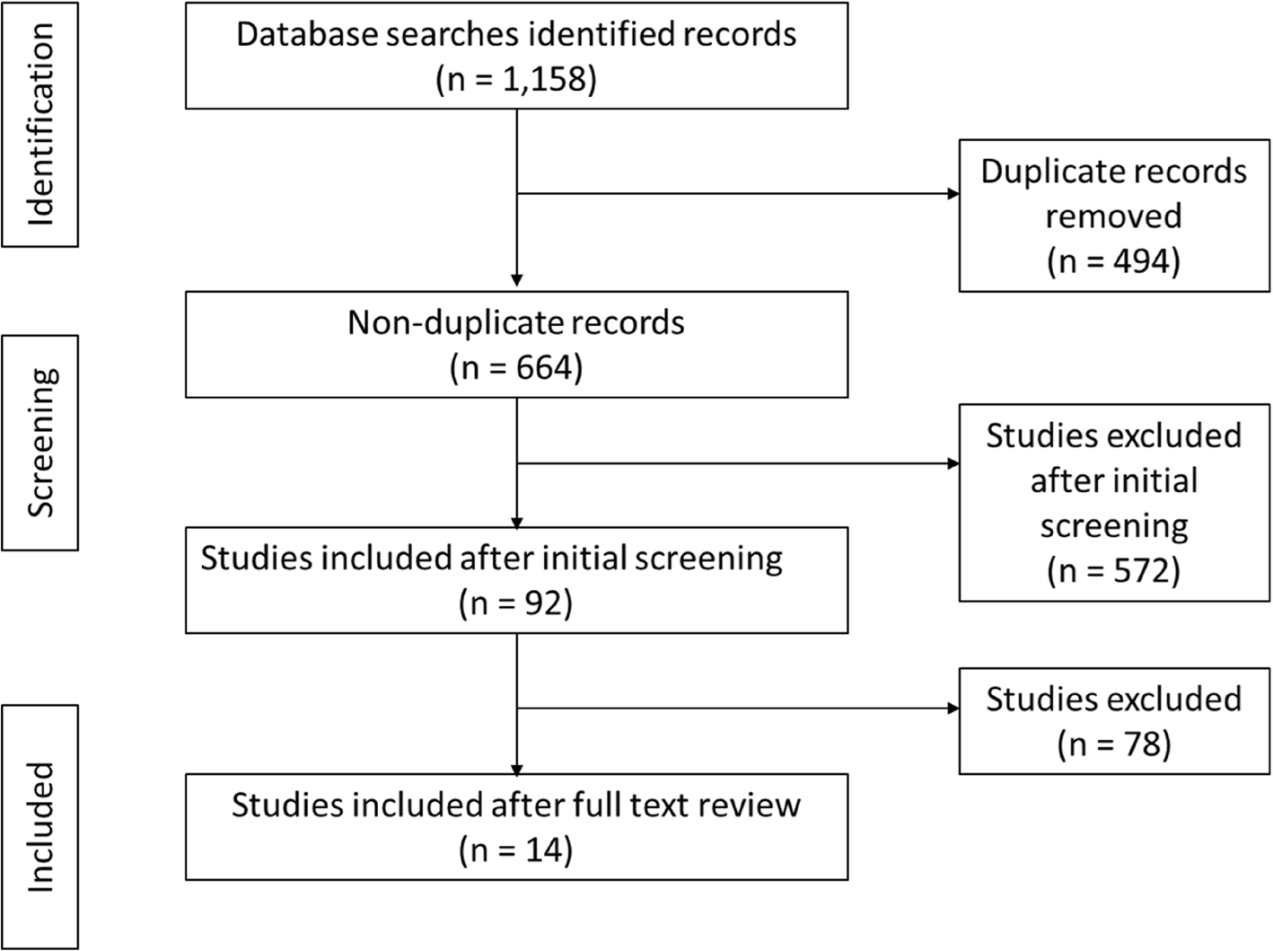
Phase one scoping review literature selection

In phase one, 1,158 articles were identified across the various databases: 276 from CINAHL; 257 from OVID; 287 from PubMed; and 338 from Web of Science. Four hundred and ninety-four duplicates were identified and removed, leaving 664 articles for review; 215 were published in 2020, 355 in 2021, and 94 in 2022. Following reviewing all articles, 572 were excluded based on assessment against inclusion and exclusion criteria and 92 were screened (Fig. 1). Following screening, 14 articles were in scope (Table 4).

**Table 4:** Characteristics of reviewed studies

Authors	Year	Title	Journal	Data collection method	Aims/ Objectives	Number of participants	Country/ region	Study outcomes relevant to health services access	Recommendations
**Phase one**									
Bailey, A., Harris, MA., Bogle, D., Jama, A., Muir, SA., Miller, S., Walters, CA., & Govia I	2021	Coping with COVID-19: Health risk communication and vulnerable groups	*Disaster Medicine and Public Health Preparedness*	Media content analysis and semi-structured interviews of key informants and individuals living with physical disabilities, mentally ill people, and the elderly	Identify communications materials contributing to improving public awareness; describe lived experiences of elderly people, those with physical disabilities, and those living with mental disorders; and describe strategies/ interventions authorities, family, and caregivers use to support vulnerable people	35 interviews in total, 10 of these with key informants	Jamaica	Much of the messaging and communications targets the general population, rather than vulnerable populations. Participants reported emotional responses towards the pandemic, including fears in accessing health services. They further suggested changes to personal risk management, and delays in accessing timely information	Official risk communication messaging through agencies supporting vulnerable people
Dai, R., & Hu L	2022	Inclusive communications in COVID-19: a virtual ethnographic study of disability support network in China	*Disability & Society*	WeChat ethnography of a single WeChat Disability Support Network group involving observation, participation, and online semi-structured interviews	Understand the impact of a volunteer driven, non-Government disability support network to support emergency responses for disabled people and understand what accessible and effective information communication looks like for disabled people	The WeChat group contained approximately 190 volunteers, half were people with disabilities or family members of disabled people, some were from outside China	China	People with disabilities need “accurate, timely, and accessible information” to be able to access healthcare (p.19). Volunteers can contribute to reaching disabled people in their community with information about healthcare	Research team recommend that people with disabilities need to be involved in health service planning and provision because without lived experience of disability interventions will not be effective in meeting the needs of those in the community
Embregts, P., Heerkens, L., Frielink, N., Giesbers, S., Vromans, L., & Jahoda, A	2021	Experiences of mothers caring for a child with an intellectual disability during the COVID‐19 pandemic in the Netherlands	*Journal of Intellectual Disability Research*	Semi-structured in-depth interviews following a convenience sampling process	Understand the experiences of parents of children with intellectual disabilities during the first COVID-19 lockdown phase	5 participants, all mothers	Netherlands	The findings from this research revealed three themes: 1. “We need to stay healthy” 2. “We make it work” and 3. “my child's and family’s place in the world”. The findings highlight the challenges for mothers during a pandemic and propose recommendations for them whilst they care for children with an intellectual disabilities	Mothers who are caring for disabled children at home during a pandemic need time away from caring responsibilities, support from education providers and coping strategies to assist them in their roles
Epstein, S., Campanile, J., Cerilli, C., Gajwani, P., Varadaraj, V., & Swenor, BK	2021	New obstacles and widening gaps: A qualitative study of the effects of the COVID-19 pandemic on U.S. adults with disabilities	*Disability and Health Journal*	Semi-structured focus groups based on type of disability (identified through a preliminary survey)	Identify how COVID-19 impacted people with disabilities	38 participants across 12 focus groups, groups included vision impairment, hearing problems, chronic illness, mental health, mobility/ physical disabilities, and cognitive/ intellectual/ developmental difficulties	United States of America	The pandemic had a significant and unequal impact on people with disabilities. A total of three overarching themes were identified from the study: “new problems created by the pandemic”, “obstacles in daily life that were exacerbated by the pandemic” and “changes to accessibility and disability identity”. Issues identified by the participants included the rationing of healthcare, disruption to disability services and challenges with accessing routine healthcare	It is important that public health providers include the voice of people with disabilities when planning health services to mitigate the unequal burden caused by the pandemic
Kim, MA., Yi, J., Sung, J., Hwang, S., Howey, W. & Jung, SM	2021	Changes in life experiences of adults with intellectual disabilities in the COVID-19 pandemics in South Korea	*Disability and Health Journal*	Semi-structured in-depth interviews with a purposive sample	Understand how adults with intellectual disabilities experienced challenges and adapted during the closure of services during the COVID-19 pandemic	15 participants with a range of mild-severe intellectual disabilities	South Korea	Five themes emerged from this study: Health behaviours, daily life, family relationships, social participation and social relationships (p.3–4). People with disabilities found ways to adapt to the burdens caused by the pandemic	The findings of this research highlight the significant impact the pandemic had for people with disabilities; they also inform health service planners seeking to support people with disabilities during pandemics, enabling a prioritisation of social healthcare and service initiatives
Kwegyir Tsiboe, A	2020	Describing the experiences of older persons with visual impairments during COVID-19 in rural Ghana	*Journal of Adult Protection*	Semi-structured in-depth interviews	Understand the lived experiences of older disabled persons in rural Ghana and determine applicable solutions	20 participants, aged between 60 and 79 years who were vision impaired	Petu, Ghana (rural)	The lockdown and the pandemic for the participant group that lived alone led to isolation, negatively impacted mental health and hunger because of loss of informal support services. For those who lived in families, the fear of a lack of healthcare in the area kept them isolated within their homes	There are valuable insights that can be gained from examining the experiences of disabled people during pandemics; there is also a need for accessible health care, especially for those in rural areas. Care provision and protection for people with disabilities should be legislated to ensure stability and improve health outcomes
Lindsay, S., Ahmed, H., & Apostolopoulos, D	2021	Facilitators for coping with the COVID-19 pandemic: Online qualitative interviews comparing youth with and without disabilities	*Disability and Health Journal*	Semi-structured in-depth interviews	Understand wellbeing facilitators for (disabled) youth and young adults during the COVID-19 pandemic	34 participants aged 16–29 years; 17 with a range of physical, mental health, and learning disabilities, 17 without disabilities. Participants were predominantly women	Ontario, Canada	The findings demonstrate that during the pandemic there were differences in the way that young people attended to their mental health dependant upon if they did or did not have a disability. Protective mechanisms included daily activities, social support, managing the balance between work-life and access to social support	The ability to address stressors caused by the pandemic were mediated through coping strategies. Young people with disabilities need additional support to help them to manage pandemic-related stressors
Mathias, K., Rawat, M., Philip, S., & Grills, N	2020	We’ve got through hard times before: acute mental distress and coping among disadvantaged groups during COVID-19 lockdown in North India—a qualitative study	*International Journal for Equity in Health*	Semi-structured in-depth interviews	Determine the acute mental health impacts of the COVID-19 crisis and coping strategies employed by disadvantaged community members	24 participants, comprising 16 people participating due to their disability or psychosocial disability (four of these interviews were with carers), and 8 widows	North India	Participants in this study had limited access to healthcare or mental health services. They experienced “intersectional disadvantage” that negatively impacted their mental health. Experiences included othering by others, racism and mental distress; despite this the participants developed strategies to counteract the inequitable treatment	To be able to deliver meaningful and responsive care to the participant group, an intersectional approach needs to be used to address the needs of people with disabilities
Reber, L., Kreschner, JM., DeShong, GL., & Meade, MA	2022	Fear, isolation, and invisibility during the COVID-19 pandemic: A qualitative study of adults with physical disabilities in marginalized communities in South-Eastern Michigan in the United States	*Disabilities*	Semi-structured in-depth interviews	Identify COVID-19-related barriers and facilitators for adults with long-term physical disabilities from marginalized communities	15 participants with moderate-severe physical disability, comprising 60% African American population. Eight participants also had other links to the disability community through activism/ employment	South-eastern Michigan, United States of America	The findings reveal that the pandemic has been impactful on the participants lives in myriad ways, including feeling invisible, isolated and alone especially when accessing health care. Authors describe the participants as 'always already vulnerable and that to have their needs met they needed to engage in behaviours that put them at risk	Issues of racism, socioeconomic inequity and ableism are structural issues that need to be tackled by healthcare systems. Healthcare policy needs to change to reflect disabled people's experiences; for example, allow caregivers to attend appointments. Qualitative research decreases the invisibility of people with disabilities and results in meaningful health policy at a local and national level
Saunders, GH., Jackson IR., & Visram, AS	2021	Impacts of face coverings on communication: an indirect impact of COVID-19	*International Journal of Audiology*	Social media driven snowball sampling survey of general public, with over sampling of those with hearing loss. Survey had both open and closed questions	Determine the impact face coverings have on hearing and communication in the period prior to face masks becoming mandatory	460 participants, 50% use cochlear implants or own hearing aids	United Kingdom	Face coverings negatively effected hearing impaired people, and visual cues were covered by mask wearing; these issues impacted the quality of interactions for hearing impaired people leading to listening fatigue and the need for strategies to counter the additional challenges	The impact of mask wearing has had significant implications for hearing impaired people. There is a need for communication friendly masks and strategies for health professionals to communicate effectively with the hearing impaired community
Schwartz, AE., Munsell, EGS., Schmidt, EK., Colón-Semenza, C., Carolan, K., & Gassner, DL	2021	Impact of COVID-19 on services for people with disabilities and chronic health conditions	*Disability and Health Journal*	Survey of adults with disabilities and chronic health conditions. Survey had both open and closed questions	Understand how service disruptions affected the daily activities and participation of disabled individuals and those with chronic health conditions, including for employment in the first several months of the COVID-19 outbreak	119 participants	United States of America	The pandemic negatively impacted the ability to access physical therapy, peer support, transportation and mental health services. Extended families helped to fill the gaps left by limited or lost service provision; however, telehealth did support ease of access to health services. Their was an increased need for mental health support	The pandemic created gaps in service provision for people with disabilities; however, telehealth meant that mental health support could continue despite lockdowns. There is a need for disability care coordination to bridge multiple services for this group. The research also additional research that highlights the needs of minority populations
Sutter, EN., Smith Francis, L., Francis, SM., Lench, DH., Nemanich, ST., Krach, LE., Sukal-Moulton, T. & Gillick, BT	2021	Disrupted access to therapies and impact on well-being during the COVID-19 pandemic for children with motor impairment and their caregivers	*American Journal of Physical Medicine & Rehabilitation*	Survey of parents of children with motor disabilities, survey included three open-ended questions into the lived experiences of these parents and their perceptions of caregiving	Determine caregiver perceptions of the pandemic’s impact on paediatric rehabilitation therapy access, caregiver satisfaction with these therapies, and physical and wellbeing outcomes for children with motor impairment and their caregivers	102 participants, 7% response rate	United States of America	The inability to attend rehabilitation/physical activity was linked to increased stress in both children and adults. The overarching themes were: Impact on access to therapies; Satisfaction with therapies; Impact of child/caregiver well-being. The impact of telehealth highlighted unique challenges and benefits, including ease of access for those from rural areas and difficulties related to not having physical access specialist therapists	COVID-19 resulted in significant service delivery; access to rehabilitate therapies for CWD has been negatively impacted by the pandemic. Online therapy delivery had positive and negative impacts on children and parents. The research demonstrates the value of rehabilitation therapy for CWD but additional research is required to explore experiences for diverse population groups
Theis, N., Campbell, N., De Leeuw, J., Owen, M., & Schenke, KC	2021	The effects of COVID-19 restrictions on physical activity and mental health of children and young adults with physical and/or intellectual disabilities	*Disability and Health Journal*	Survey of parents/ caregivers of children with disabilities, survey included open-ended questions	Investigate physical activity and mental health changes in children and young adults with physical and/or intellectual disabilities because of COVID-19 lockdown measures	125 participants, ~ 31% response rate	United Kingdom	Lockdowns because of COVID-19 negatively impact their children’s mental health and ability to be physically active. In part this was because special accessible facilities and support services for disabled people were not available during the pandemic. The impact of these restrictions will result in additional needs for disabled people at a level above what was required pre-pandemic	Access to physical activity facilities and mental health services needs to be reviewed to meet the additional needs of disabled people as lockdowns are eased. There is also a need to assess the social needs of people with disabilities and ensure that adequate support and mechanisms for engagement are offered
Xu, D., Yan, C., Zhao, Z., Weng, J., & Ma, S	2021	External communication barriers among elderly Deaf and hard of hearing people in China during the COVID-19 pandemic emergency isolation: A qualitative study	*International Journal of Environmental Research and Public Health*	Semi-structured in-depth interviews	Understand the effect of the COVID-19 outbreak on older deaf and hard of hearing people during COVID-19 emergency quarantine, specifically focussed on external communication needs for living and medical care	13 participants with hearing disabilities, aged over 60 years located in urban locations within the province	Wuhan, China	To explore how COVID-19 and associated lockdown impacted the deaf and hard of hearing community	Communication with the deaf and hard of hearing community needs to be bolstered during a pandemic, through additional support for social and disability services. Any activities need to take into account the vulnerabilities in the disabled community
**Phase two**									
AlMeraj, Z., Abu Doush, I., Alhuwail, D., Shama, S., AlBahar, A., & Al-Ramahi, M	2023	Access and experiences of Arabic native speakers with disabilities on social media during and after the world pandemic	*International Journal of Human–Computer Interaction*	Social media posts and semi-structured interviews	Assessing the accessibility of information disseminated by local government on social media about COVID-19, and the experiences of disabled people during lockdowns	18	Kuwait	Social media information is not fully accessible to people who are vision impaired, blind, deaf or hearing impaired	Accessibility evaluations should form a part of the information for communities. A variety of social media channels should be employed to ensure accessibility. Legal protection for disabled people should govern the information created and disseminated by governments
Arbour-Nicitopoulos, KP., James, ME., Moore, SA., Sharma, R., & Martin Ginis, KA	2022	Movement behaviours and health of children and youth with disabilities: Impact of the 2020 COVID-19 pandemic	*Paediatrics & Child Health*	Online survey and interviews	Investigated the short and long-term impacts of the pandemic on the health and movement of disabled young people and children	Interview *n* = 7, Survey *n* = 86	Canada	Children and young people were less active and reported poorer health outcomes as a result of the pandemic; this was attributed to changes in routines, access to physiotherapy, and social environments	There is a need to focus on and prioritise the health and movement of this population group and for highly trained personnel to support families
Arichi, T., Cadwgan, J., McDonald, A., Patel, A., Turner, S., Barkey, S., Lumsden, DE., & Fairhurst, C	2022	Neurodisability care in the time of COVID-19	*Child: Care, Health and Development*	Survey	Explore the impact of the pandemic’s initial phases on disabled young persons’ and children’s their social provision, education and health	*n* = 108	United Kingdom	Lockdowns were challenging for families and impacted stress levels. Children experienced a loss of therapy and clinical appointments	The pandemic has had a disproportionate impact on children and young people with neurodisabilities; this study highlights gaps in current service provision during public health emergencies. Research is needed to ensure these populations are supported in the future through improved emergency planning
Bellon, M., Idle, J., Lay, K., & Robinson, S	2022	Community in the pandemic: experiences and strategies by people with acquired brain injury and their families during COVID-19	*Disability and Rehabilitation*	Semi-structure interviews	Investigate the experiences of people with brain injury and their families and identify strategies for improving community connections and service provision	*n* = 16	Australia	Participants were isolated, routines were disrupted and people developed strategies for staying healthy and safe	There is a need to provide accessible information, and technology; it is also important to connect with families during pandemics. Peer/support networks are also vital for this population group
Bergmans, RS., Chambers-Peeple, K., Aboul-Hassan, D., Dell’Imperio, S., Martin, A., Wegryn-Jones, R., Xiao, LZ., Yu, C., Williams, DA., Clauw, DJ., & DeJonckheere, M	2022	Opportunities to improve long COVID care: implications from semi-structured interviews with Black patients	*The Patient—Patient-Centered Outcomes Research*	Semi-structure interviews	The development of strategies for healthcare delivery that are responsive for African Americans with long-COVID	*n* = 39	United States	General practitioners were the first source of healthcare sought to assist with long-COVID; however, participants did not always receive the help they needed, which impacted their ability to receive support services resulting in additional hardship	There is a need for care plans, services that are responsive to African Americans. Primary healthcare needs education to be able to responsively care for this cohort
Binder-Olibrowska, KW., Wrzesińska, MA., & Godycki-Ćwirko, M	2022	Is telemedicine in primary care a good option for Polish patients with visual impairments outside of a pandemic?	*International Journal of Environmental Research and Public Health*	Survey	Assess the interest of disabled people in accessing telemedicine during the pandemic	219	Poland	Half the respondents were interested in using telemedicine. The respondents' age was closely linked to the desire to access telemedicine	Primary healthcare should offer telemedicine to people with vision impairment. Staff should be aware of communication guidelines for vision-impaired people and have access to appropriate technology to support communication. People with visual impairments should be involved in developing care strategies
Bozkus-Genc, G., & Sani-Bozkurt, S	2022	How parents of children with autism spectrum disorder experience the COVID-19 pandemic: Perspectives and insights on the new normal	*Research in Developmental Disabilities*	Semi-structure interviews	Examine pandemic experiences of parents with children who have autism spectrum disorder	*n* = 8	Turkey	Parents experienced stress related to lockdowns, which increased over time. They expressed mental health challenges and issues with the distance education system	Parents with disabled children need additional support during the pandemic to support their family functioning, and their child's wellbeing and overall health. The findings of this research reinforce the need for revising current health and disability service provision to ensure optimal outcomes for this cohort in the future
Burke, MM., Cheung, WC., Li, C., DaWalt, L., Segal, J., & Taylor, JL	2022	Parental perceptions of service access for transition-aged youth with autism during COVID-19	*Intellectual and Developmental Disabilities*	Structured interviews	Explore experiences of parents in accessing services for their young person with autism spectrum disorder during the pandemic	*n* = 65	United States	None of the participants were able to access new services during the pandemic. Often services were moved to telehealth at the behest of practitioners, not families	Service disruption had a significant impact on families; flexibility in service provision is one strategy for overcoming some of the challenges. Equity needs to be considered when delivering services to this population. The effectiveness of telehealth for this cohort needs further research
Buse, DC., Gerstein, MT., Houts, CR., McGinley, JS., Uzumcu, AA., McCarrier, KP., Cooke, A., Touba, NM., Nishida, TK., Wirth, RJ., & Lipton, RB	2022	Impact of the COVID-19 pandemic on people living with migraine: Results of the MiCOAS qualitative study	*Headache*	Semi-structure interviews	Examine the impact of the pandemic with people living with migraines	40	United States	The pandemic led to negative and positive impacts for migraine suffers. Telehealth potentially offers this cohort better access to healthcare during migraine attacks	Focusing on lifestyle factors can offer positive impacts for those with migraines. Further, telehealth offers an option for improved access to healthcare for this cohort
Caldwell, J., Heyman, M., Atkins, M., & Ho, S	2022	Experiences of individuals self-directing Medicaid Home and Community-Based Services during COVID-19	*Disability and Health Journal*	Semi-structure interviews	Investigate how participants accessed self-directed disability services, maintained their safety and managed their health	36	United States	Flexibility came with benefits in relation to managing healthcare workers but there were service disruptions because of COVID-19. The flexibility meant family members could step into the role. Healthcare was disrupted and delayed but technology enabled different approaches to accessing care	Flexibility in service funding and provision enabled participants to manage their needs. Planning around health services during pandemics is vital for the disabled population
Chaiban, L., Benyaich, A., Yaacoub, S., Rawi, H., Truppa, C., & Bardus, M	2022	Access to primary and secondary health care services for people living with diabetes and lower-limb amputation during the COVID-19 pandemic in Lebanon: a qualitative study	*BMC Health Services Research*	Semi-structure interviews	Investigate the barriers to healthcare access for those with lower-limb amputations	8	Lebanon	Research revealed barriers related to financial costs, transport, medication, ableism, coupled with fear of contracting COVID-19	Significant barriers to healthcare have resulted in reduced access that requires a system response. There is a need for equitable access to health services for disabled people. Gender equity is also important; this should be the focus of additional research
Chirico, I., Ottoboni, G., Giebel, C., Pappadà, A., Valente, M., Degli Esposti, V., Gabbay, M., & Chattat, R	2022	COVID-19 and community-based care services: Experiences of people living with dementia and their informal carers in Italy	*Health & Social Care in the Community*	Semi-structure interviews	Explore the experiences of people with dementia and their carers who attend community services	22	Italy	The pandemic has disrupted the lives of people with dementia, leading to stress for carers because of the additional care load. Carers created innovative solutions to support social care	Social services need to take into account the needs of those with dementia. There is a need to focus on holistic care provision
Chowdhury, S., Urme, S. A., Nyehn, BA., Mark, HR., Hassan, MT., Rashid, SF., Harris, NB., & Dean, L	2022	Pandemic portraits—an intersectional analysis of the experiences of people with disabilities and caregivers during COVID-19 in Bangladesh and Liberia	*Social Sciences*	Photovoice	Explore the experiences of the pandemic for those with disabilities and their caregivers	27	Bangladesh	The results highlighted the inaccessibility of community spaces, social connections, and explored the adaptability, fears and hopes of the participant group	This research demonstrates that photovoice facilitates the emergence of insights into how to create pandemic responses that are accessible and inclusive
Cochran, AL., McDonald, NC., Prunkl, L., Vinella-Brusher, E., Wang, J., Oluyede, L., & Wolfe, M	2022	Transportation barriers to care among frequent health care users during the COVID pandemic	*BMC Public Health*	Open-ended survey	Assess the transportation barriers for those with chronic illnesses accessing healthcare	323	United States	One third of respondents struggled to access healthcare during the pandemic. Those without a car or with disabilities are more likely to struggle to access healthcare	There is a need for a coordinated response to address the transportation needs of high-risk groups to access healthcare. The response should address health needs, including financial support to support attending healthcare appointments
Costa, B., McWilliams, D., Blighe, S., Hudson, N., Hotton, M., Swan, MC., & Stock, NM	2021	Isolation, uncertainty and treatment delays: Parents’ experiences of having a baby with cleft lip/palate during the COVID-19 pandemic	*The Cleft Palate Craniofacial Journal*	Semi-structure interviews	Explore the pandemic’s impact on parents with infants who have a cleft lip/palate	14	United Kingdom	The pandemic resulted in reduced contact between parents and healthcare providers and changed healthcare provision, because of this parents were anxious. In addition, surgery was often delayed leading to ambiguity for families	Parents need access to healthcare professionals that can ease the burden of caring for these infants. Social support is one mechanism that can assist to alleviate stress
Currie, G., Finlay, B., Seth, A., Roth, C., Elsabbagh, M., Hudon, A., Hunt, M., Jodoin, S., Lach, L., Lencucha, R., Nicholas, DB., Shakako, K., & Zwicker, J	2022	Mental health challenges during COVID-19: perspectives from parents with children with neurodevelopmental disabilities	*International Journal of Qualitative Studies on Health and Well-being*	Interview	Examined the impacts of pandemic restrictions on mental health of parents and their children who have neurodevelopmental disorders	40	Canada	Parents experienced mental health challenges due to gaps in support systems. Inability to access disability and health services negatively impacted family functioning	Inclusive approaches are needed to support families with disabled children; this needs to include resources for families to support them in the community and at home
Dean, NA., Marwaha, A., Grasdal, M., Leong, S., Mesa, A., Krassioukov, AV., & Bundon, A	2022	Perspectives from the spinal cord injury community with teleSCI services during the COVID-19 pandemic: A qualitative study	*Disability and Rehabilitation: Assistive Technology*	Interview	Investigate the experiences of those with spinal cord injury using teleSCI services during the pandemic	12	Canada	Participants indicated that teleSCI was an affordable, accessible, and convenient approach to accessing care during the pandemic; however, in-person assessments were still required and should be available	Flexibility in the mode of service provision is warranted
Dodds, RL., Maurer, KJ., Montgomery, LS., Cutting, S., & Jilek, C	2022	Self-advocate perspectives on COVID-19 in Urban Los Angeles: impacts on autonomy and access to supports	*Journal of Intellectual & Developmental Disability*	Interview	Understand the experience of those with intellectual and developmental disabilities during the pandemic	14	United States	Autonomy is important for the participant group, but this requires the ability to make choices, link with family, disability services and access their daily living needs	People with intellectual and developmental disabilities require education to use technology to access healthcare, social support and disability services
Filbay, S., Bennell, K. L., Morello, R., Smith, L., Hinman, R. S., & Lawford, BJ	2022	Exploring experiences with telehealth-delivered allied healthcare services for people with permanent and significant disabilities funded through a national insurance scheme: a qualitative study examining challenges and suggestions to improve services	*BMJ Open*	Interview	Investigate the challenges that people with disabilities or their carers had accessing telehealth during the pandemic	12	Australia	The difficulties identified by participants included the need for carer facilitation, challenges with trust and clinician feedback coupled with access challenges and lack of engagement on the part of the person with disability	There is a need to assess the suitability of and increase exposure to telehealth and plan and manage expectations
Filler, T., Benipal, P. K., Minhas, R. S., & Suleman, S	2022	Exploring the impact of COVID-19 on families of children with developmental disabilities: A community-based formative study	*Paediatrics & Child Health*	In-depth interview	Investigate the experiences of families of children with developmental disabilities during the pandemic	25	Canada	Participants revealed that social isolation requirements were challenging to adhere to, which resulted in parents being stressed. Families were further stretched by financial challenges and the inability to access services	The participants were negatively impacted by the pandemic. Continued access to services is imperative to mitigate the negative impact of such events
Forslund, T., Fernqvist, S., & Tegler, H	2022	Parents with intellectual disability reporting on factors affecting their caregiving in the wake of the COVID-19 pandemic: A qualitative study	*Journal of Applied Research and Intellectual Disability*	Semi-structure interviews	Explore the impact of the pandemic on parents with intellectual disabilities	10	Sweden	Reduced resources and increased carer demands stressed parent–child dyads. Without adapted information, a lack of informal educational and disability support parents struggled	Parents need access to support in the form of stress-release strategies, adapted materials and contextual models that account for their unique needs
Fridell, A., Norrman, H. N., Girke, L., & Bölte, S	2022	Effects of the early phase of COVID-19 on the autistic community in Sweden: A qualitative multi-informant study linking to ICF	*International Journal of Environmental Research and Public Health*	Interview	Explore the experiences of the autistic community during the pandemic	38	Sweden	Participants report that the impact of the pandemic on their lives was significant; leading to increased social isolation, disrupted education, and reduced access to healthcare, as a result, their mental health was negatively impacted	Participants recorded health impacts during the pandemic, such as mental health challenges, loss of service support and concerns about contracting COVID-19. They also reported strategies for maintaining health despite lockdowns, which included staying active, connecting and using technology
Goddard, K. S., Schulz, J., Nzuki, I., & Hall, J. P	2022	Examining the impacts of the coronavirus pandemic and social distancing on the health of people with mobility disabilities	*Frontiers in Public Health*	Open-ended survey	Investigate the health impacts of the pandemic on those with mobility disabilities	39	United States	Changes to health services, access to disability services, and a lack of accessible transportation contributed to disabled people experiencing adverse outcomes due to the COVID-19 pandemic	Policies have not been designed to meet the needs of disabled people, which have negatively impacted this cohort during the pandemic
Good, G., Nazari Orakani, S., Officer, T., Roguski, M., & McBride-Henry, K	2022	Access to health and disability services for blind New Zealanders during the COVID-19 pandemic 2020–2022	*Journal of Visual Impairment & Blindness*	Interview	Explore the experiences of vision impaired New Zealanders during the pandemic	10	Aotearoa, New Zealand	The pandemic had a negative impact on the vision impaired community; these included negative impacts on mental health, social isolation, a loss of practical support. Transport challenges because of a loss of disability services were also noted	Vision impaired people need to be involved in creating solutions for future pandemics
Goodley, D., Lawthom, R., Liddiard, K., & Runswick-Cole, K	2022	Affect, dis/ability and the pandemic	*Sociology of Health & Illness*	Blog	Explore the impacts of the pandemic on disabled people	22 blogs from 15 countries	United Kingdom	The pandemic highlighted the fragility of disabled people, and the anxiety they experienced as a result. It also highlighted the emergence of support groups and online support systems	Disabled people need to be positioned at the centre of any initiatives to improve recovery post-pandemic
Govia, I., Palmer, T., Stubbs, M., Harris, M., Bogle, D., Miller, S., Walters, C., Muir, S. A., & Bailey, A	2022	Vulnerable group members coping with COVID-19 in Jamaica: A qualitative study	*Traumatology*	Semi-structure interviews	Understand the experience of vulnerable people groups as a result of COVID-19	25	Jamaica	Participants' experiences highlight the vulnerability of these groups, with issues raised around service gaps, unmet needs and mental distress because of the rationing of services	There is a need to include vulnerable groups in disaster planning and focus on developing research that addresses their needs
Gul, S., & Ygmur, Y	2022	The access of women with disabilities to reproductive health services during the COVID-19 pandemic: A qualitative study	*International Journal of Caring Sciences*	Semi-structure interviews	Explore disabled women’s experience of accessing reproductive health services during the pandemic	28	Turkey	Disabled women struggled to access reproductive health services during the pandemic. They highlighted the important of timely and meaningful access as well as the challenges they experienced	Maintaining access to reproductive services is important for this cohort. Nurses are in a prime position to support such initiatives
Hall, K. A. E., Deusdad, B., D’Hers Del Pozo, M., & Martínez-Hernáez, Á	2022	How did people with functional disability experience the first COVID-19 lockdown? A thematic analysis of YouTube comments	*International Journal of Environmental Research and Public Health*	Comments posted to a YouTube channel	Explore the narratives of those with functional disabilities from posts to a YouTube video during a pandemic lockdown	100 comments	Spain	Comments highlighted social isolation and a lack of access to services, which resulted in mental health impacts	The research exposes inadequate services for those with functional disabilities even in nations with developed social service systems
Hielscher, L., Ludlow, A., Mengoni, S. E., Rogers, S., & Irvine, K	2022	The experiences of new mothers accessing feeding support for infants with Down syndrome during the COVID-19 pandemic	*International Journal of Developmental Disabilities*	Semi-structure interviews	Impact of the pandemic on mothers of infants with Down syndrome	13	United Kingdom	Each infant and mother dyad has a unique journey; health professionals lack knowledge, which causes frustration for mothers; additional support is needed to support infant feeding	Individualised support is needed for mothers of Down syndrome infants
Hochman, Y., Shpigelman, C.-N., Holler, R., & Werner, S	2022	“Together in a pressure cooker”: Parenting children with disabilities during the COVID-19 lockdown	*Disability and Health Journal*	Open-ended survey	Parents’ experiences of lockdown with their disabled children	80	Israel	Analysis revealed positive and negative challenges that are primarily focused on the families’ support needs; parents report these are focused on education and social services	The social model of disability enables robust analysis of family experiences. A lack of targeted policies left families in challenging positions
Isensee, C., Schmid, B., Marschik, P. B., Zhang, D., & Poustka, L	2022	Impact of COVID-19 pandemic on families living with autism: An online survey	*Research in Developmental Disabilities*	Open-ended survey	Investigate the impact of the pandemic on families and their children who have autism spectrum disorder	216	Germany	Half of the respondents indicated their child’s symptoms worsened during the pandemic, which was linked to increases in parental stress and inaccessible therapy; this resulted in increased medication for the children	The pandemic has resulted in negative impacts on children with autism spectrum disorder. Additional research is required to understand the long-term impacts on this population
LaVela, S. L., Wu, J., Nevedal, A. L., Harris, A. H. S., Frayne, S. M., Arnow, K. D., Barreto, N. B., Davis, K., & Eisenberg, D	2022	The impact of the COVID-19 pandemic on individuals living with spinal cord injury: A qualitative study	*Rehabilitation Psychology*	Semi-structured interviews	Explore the impact of the pandemic on people with spinal cord injuries	33	United States	The pandemic had various impacts on the participant group; these included, disability services, access to therapy and healthcare, lifestyle, social interactions and independence	The impact of the pandemic was significant and wide-ranging on the population group; the findings reveal myriad gaps in service provision that negatively impact the health and wellbeing of this cohort. This information can be used to inform health services seeking to develop responsive public health measures for future pandemics
Linden, M. A., Forbes, T., Brown, M., Marsh, L., Truesdale, M., McCann, E., Todd, S., & Hughes, N	2022	Impact of the COVID-19 pandemic on family carers of those with profound and multiple intellectual disabilities: Perspectives from UK and Irish Non-Governmental Organisations	*BMC Public Health*	Focus groups	Explore the impact of the pandemic on carers of children with intellectual disabilities	24	Republic of Ireland and United Kingdom	Participants raised issues around mental distress, isolation, fear and exhaustion. They also indicated they had a lack of trust in formal services and discussed online support	The lack of access to support and services to assist in caring for children was exacerbated during the pandemic. There is an immediate need to build meaningful services that include parents in their design
Mazzoni, N., Bentenuto, A., Filosofi, F., Tardivo, A., Strathearn, L., Zarei, K., De Falco, S., Venuti, P., Iandolo, G., & Giannotti, M	2023	Parenting a child with a neurodevelopmental disorder during the early stage of the COVID-19 pandemic: Quantitative and qualitative cross-cultural findings	*International Journal of Environmental Research and Public Health*	Survey	Understand the extent of the impact on the symptoms of children with neurodevelopmental disorders, cessation of therapy and parental stress before and during the pandemic	1494	United States	Regardless of geographic location parental stress increased during the pandemic; this increase was attributed to the lack, or discontinuation, of therapy. Parental efficacy and resilience positively impacted stress levels	Strategies for increasing resilience might reduce the levels of stress in parents and families
Mbazzi, F. B., Nalugya, R., Kawesa, E., Nimusiima, C., King, R., van Hove, G., & Seeley, J	2022	The impact of COVID-19 measures on children with disabilities and their families in Uganda	*Disability & Society*	Interview	Explore the impact on families with disabled children because of the pandemic	39	Uganda	Parents indicated the pandemic had multifaceted impacts on them and their disabled children. Access to healthcare had been reduced or removed, with concerns for the basic life necessities	Comprehensive service responses are needed to meet the needs of families with disabled children. Such responses should be based on this group's involvement
Mitwalli, S., Kiwan, D., Abdul-Samad, L., & Giacaman, R	2022	The double burden of COVID-19 and Israeli military rule on persons with disabilities in the West Bank of the occupied Palestinian territory	*Frontiers in Psychology*	Interview	Explore the impact on families with disabled children in the West Back as a result of the pandemic	16	Palestinian National Authority	Participants reported that the pandemic had significantly impacted their lives. Previous challenges across many areas were exacerbated, including disability services and healthcare access	The results highlight the challenges and barriers for people with disabilities that resulted from the pandemic. People with disabilities need to be central to developing solutions that meet their needs
Mohamed, H., Wamera, E., & Malima, W	2022	Access to Water, Sanitation and Hygiene Services and other preventive measures against COVID-19 among people with disabilities, Dodoma, Tanzania	*American Journal of Tropical Medicine and Hygiene*	Interview and focus group	Explore disabled people’s access to pandemic prevention initiatives and adequate Water, Sanitation and Hygiene (WASH)	102	Tanzania	Disabled people did not have access to education about, WASH or other COVID-19 prevention initiatives. The most affected by this were those with physical disabilities including vision and hearing impairments	Disabled people need to be positioned at the centre of any initiatives to improve recovery post-pandemic
Navas, P., Verdugo, M. Á., Martínez, S., Amor, A. M., Crespo, M., & Deliu, M. M	2022	Impact of COVID-19 on the burden of care of families of people with intellectual and developmental disabilities	*Journal of Applied Research in Intellectual Disabilities*	Survey	Investigate the carer burden in families with members with developmental and intellectual disability	323	Spain	The stress level amongst the respondents increased during the pandemic, which was attributable to the care burden and loss of disability services	Authors call for support services to continue through future pandemics to ensure family wellbeing
Nguyen, L., & Bui, M	2022	Social protection response to COVID-19: Experiences and lessons from Vietnam	*Asia Pacific Journal of Social Work and Development*	Interview	Understand the impact of the COVID-19 pandemic on people in light of social protection policies that aimed to protect community groups	58	Vietnam	Vietnam had many social services in place prior to the pandemic; these met some needs during pandemic restrictions, but disabled people reported that there were many gaps in service provision leading them to rely heavily on neighbours and extended family. Lessons can be learned from Vietnam's response and the pre-existing social strategies employed to support vulnerable people within the community	Communities impacted by government strategies must be involved in creating and shaping these; in this way, maximum benefit can be gained by vulnerable community groups
Nicholas, D. B., Zulla, R. T., Conlon, O., Dimitropoulos, G., Urschel, S., Rapoport, A., Katz, S. L., Bruce, A., West, L. J., Belletrutti, M., Cullen, E., & Zwaigenbaum, L	2022	Mental health impacts of the COVID-19 pandemic on children with underlying health and disability issues, and their families and health care providers	*Paediatrics & Child Health*	Structured interview	Explore the mental health consequences for families and children with health and disability challenges as a result of the pandemic	262	Canada	Negative mental health impacts were reported by participants. These included burnout, carer load and distress. A lack of parental capability and capacity to respond to all the family needs caused distress	Future pandemic planning needs to take into account the experiences of families as a result of COVID-19. There is a need for proactive policies and capacity building to protect families in similar situations in the future
Oude Lansink, I. L. B., van Stam, P. C. C., Schafrat, E. C. W. M., Mocking, M., Prins, S. D., Beelen, A., Cuppen, I., van der Pol, W. L., Gorter, J. W., & Ketelaar, M	2022	‘This battle, between your gut feeling and your mind. Try to find the right balance’: Parental experiences of children with spinal muscular atrophy during COVID-19 pandemic	*Child: Care, Health and Development*	Semi-structured interview	Understand the experience of parents with children with spinal muscular atrophy during the pandemic	19	Netherlands	Parents highlighted they felt they were balancing resilience, vulnerability and security during the pandemic, but strove to protect their child during this time. They discussed their needs for information to keep their children healthy	Healthcare practitioners need to create space to share information and connect with parents; this approach will support parents to feel less vulnerable and increase a sense of agency
Pellicano, E., Brett, S., den Houting, J., Heyworth, M., Magiati, I., Steward, R., Urbanowicz, A., & Stears, M	2022	COVID-19, social isolation and the mental health of autistic people and their families: A qualitative study	*Autism*	Semi-structured interview	Explore the impact of the pandemic on autistic people’s mental health and social isolation	144	Australia	Participants described their dissatisfaction with telehealth services, preferring in-person mental health services. The pandemic also led to feelings of social isolation, causing mental distress	Autistic people need social contact and the pandemic has negatively impacted their social connections, resulting in isolation
Pincock, K., Jones, N., Baniodeh, K., Iyasu, A., Workneh, F., & Yadete, W	2022	COVID-19 and social policy in contexts of existing inequality: Experiences of youth with disabilities in Ethiopia and Jordan	*Disability & Society*	Semi-structured interview	Explore the impact of the pandemic on disabled young people in low to middle-income countries	45	Ethiopia	The pandemic has had wide-ranging negative impacts on the study cohort. Current policies are inadequate to meet the needs of disabled young people, leading to inequities	The most vulnerable within this cohort need targeted and comprehensive support
Pinkerton, L. M., Murphy, A., Bruckner, E., & Risser, H	2022	Therapy service delivery for children with disabilities during COVID-19: Parent perceptions and implementation recommendations	*Journal of Community Psychology*	Open-ended survey	Investigate the impacts of service interruptions for disabled children and young people because of COVID-19	171	United States	Families experienced disruption to services, but telehealth assisted to improve access; however, children did not always respond well to this therapy approach and parents raised issues with the use of technology during therapy	Shorter more frequent telehealth appointments were recommended by parents. Parents should form key members of any service redesign team
Portillo-Aceituno, A., Calderón-Bernal, A., Pérez-Corrales, J., Fernández-de-Las-Peñas, C., Palacios-Ceña, D., & Güeita-Rodríguez, J	2022	The impact of digital physical therapy during COVID-19 lockdown in children with developmental disorders: A qualitative study	*Brazilian Journal of Physical Therapy*	Semi-structured interviews	Explore the impact of digital therapy on children with developmental disorders	16	Spain	Parents felt ambivalent about digital therapy because it was less effective but facilitated service access for their children. Some of the challenges included keeping children engaged and focused whilst dealing with the home environment	Digital therapy could be used to complement in-person therapy, but recommendations for service delivery are offered. These include, sharing information about the purpose of this approach to care delivery for both clinicians and parents. Future research needs to explore how to optimise this digital therapy given the challenges of successfully delivering such programmes
Roguski, M., Officer, T., Nazari Orakani, S., Good, G., Händler-Schuster, D., & McBride-Henry, K	2022	Ableism, human rights, and the COVID-19 pandemic: Healthcare-related barriers experienced by Deaf people in Aotearoa New Zealand	*International Journal of Environmental Research and Public Health*	Semi-structured interview	Investigate the experiences of Deaf people when accessing healthcare	11	Aotearoa, New Zealand	Deaf people struggled to understand healthcare workers because of mask use; a failure to recognise the Deaf culture and ableist assumptions led to inequitable access to healthcare. Care provision breached the United Nations Convention on the Rights of Persons with Disabilities (CRPD)	The CRPD should guide health services and their development. Healthcare workers need training to develop competencies in working with Deaf people
Rohn, E. J., Hearn, J. H., Philippus, A. M., & Monden, K. R	2022	“It’s been a double-edged sword”: An online qualitative exploration of the impact of COVID-19 on individuals with spinal cord injury in the US with comparisons to previous UK findings	*The Journal of Spinal Cord Medicine*	Open-ended online survey	Explore the pandemic’s impact on people with spinal cord injury	36	United States	Participants faced issues accessing healthcare, managing day-to-day life and creating meaning. The findings were compared against UK respondents	The authors offer suggestions for assisting the cohort to create ways to cope with and manage the distress they experienced. Recommendations for practitioners include the need for quality therapeutic relationships and facilitating social connections within the community
Sage, R., Standley, K., & Ipsen, C	2022	“Everything is a mess. I’m just trying to survive It.”: Impacts of COVID-19 on personal assistance services	*Journal of Health Care for the Poor Underserved*	Open-ended survey	Investigate the influence of the pandemic on personal assistant services for disabled people	1638	United States	Lack of access to home care services significantly impacted disabled respondents. They raised issues related to an inability to access healthcare, basic living requirements, funding and fears about contracting COVID-19. Those with intersecting vulnerabilities were more impacted	The unmet care needs during the pandemic were significant. Disabled people continue to suffer from ongoing consequences of interruption to service provision; this negatively impacts their ability to access healthcare. Policy initiatives incorporating flexibility around service investment need to be developed to alleviate suffering
Saketkoo, L. A., Jensen, K., Nikoletou, D., Newton, J. J., Rivera, F. J., Howie, M., Reese, R. K., Goodman, M., Hart, P. B., Bembry, W., Russell, A., Lian, I., Lammi, M. R., Scholand, M. B., & Russell, A.-M	2022	Sarcoidosis illuminations on living during COVID-19: Patient experiences of diagnosis, management, and survival before and during the pandemic	*Journal of Patient Experience*	Interview	Explore the influence of COVID-19 on those with sarcoidosis in three cities	28	United States	Ableist attitudes, inequity and healthcare disruption created concerns about participant's ability to survive COVID-19. However, they reported that they had hope that the attention directed to understand multisystem respiratory disease might result in gains for the community	Results identify multifaceted issues that need to be addressed by systemic structural changes. Those with sarcoidosis could offer expertise to those with long-COVID on coping with a life transforming diagnosis
Sarica, A. D., Ulu-Ercan, E., & Coşkun, U. H	2022	COVID-19 and Turkish university students with visual impairments: An in-depth inquiry	*Journal of Visual Impairment & Blindness*	Semi-structured interview	Study the impact of the pandemic on university students with visual impairments concerning social and physical health, wellbeing and study	19	Turkey	Participants reported the pandemic impacted their daily life and created social and psychological changes. They described challenges accessing healthcare and a decline in physical activity	Universities need to be more inclusive of people with vision impairments; counsellors would be one approach to meeting their needs
Sarker, D., Shrestha, S., & Tamang, S. K. B	2022	“We'll starve to death”: The consequences of COVID-19 over the lives of poor people with disabilities in rural Nepal	*Asian Social Work and Policy Review*	Semi-structured interview	Understand the ramifications of the pandemic on disabled people in Nepal	20	Nepal	The pandemic compounded vulnerability for this participant group. Service disconnection, loss of income, isolation from healthcare and education were issues for these people	There is an urgent need for support for people with disabilities in Nepal; it needs to be a multi-system and multi-organisational approach. There is a need to protect the rights of disabled people under the CRPD
Scherer, N., Wiseman, P., Watson, N., Brunner, R., Cullingworth, J., Hameed, S., Pearson, C., & Shakespeare, T	2022	‘Do they ever think about people like us?’: The experiences of people with learning disabilities in England and Scotland during the COVID-19 pandemic	*Critical Social Policy*	Interviews	Understanding how people with learning disabilities have experienced the pandemic	24	England	Findings reveal that inequalities exacerbated the challenges associated with the pandemic; these included lack of disability services, loss of daily routines and inability to access vaccinations in a timely manner. Findings are understood using a vulnerability framework	Policies and government structures exacerbate disabled people's vulnerability and position them as marginalised within these communities
Sebring, J. C. H., Capurro, G., Kelly, C., Jardine, C. G., Tustin, J., & Driedger, S. M	2022	“None of it was especially easy”: improving COVID-19 vaccine equity for people with disabilities	*Canadian Journal of Public Health*	Focus group	Explore ways to improve equity for disabled people concerning COVID-19 vaccination	38	Canada	Barriers to accessing vaccinations related to physical accessibility of venues, the experience of receiving vaccinations, and information about vaccination and booking processes	Disabled people need to be positioned at the centre of any initiatives to improve access to vaccinations. Recommendations stemming from this research include information in accessible formats, enhanced booking systems, wheelchair accessibility, chairs available and transport. Public health responses should collaborate with disabled people to meet their needs
Selick, A., Bobbette, N., Lunsky, Y., Hamdani, Y., Rayner, J., & Durbin, J	2022	Accessibility of virtual primary care for adults with intellectual and developmental disabilities during the COVID-19 pandemic: Qualitative study	*JMIR Formative Research*	Semi-structured interview	Explore the accessibility of telehealth for those with developmental or intellectual disabilities	38	Canada	Participants reveal both positive and negative aspects of virtual care provision	Virtual health can be a useful tool for practitioners; however, an individualised approach that reflects patients' unique social, contextual, carer and healthcare needs is warranted
Sellmaier, C., & Kim, J	2023	Working and caring for a disabled adopted child during a pandemic	*Child & Family Social Work*	Survey	Explore the pandemic's impact on adoptive parents of disabled children	200	United States	Over half the respondents reported it was “somewhat” or “very difficult” to combine family and work requirements. Flexible workplaces mitigated some of this pressure. Access to disability services and mental health support helped participants	Social services and workplaces require responsive policies to support these families and adoptive parents
Sharma, Y., Whiting, A., & Dutta, T	2023	A Survey of the challenges faced by individuals with disabilities and unpaid caregivers during the COVID-19 pandemic	*International Journal of Environmental Research and Public Health*	Survey	Explore the impact of the pandemic on unpaid carers and disabled people	111	Canada	Disabled people experienced social isolation and declining physical and mental health because of an erosion of disability services. Carers reported feeling fatigued, financial impacts related to the pandemic and significant mental burdens associated with their responsibility	Increasing awareness of the experiences of caregivers and disabled people is essential. Policy suggestions include addressing assistance programmes to meet the needs of this community better. Key recommendations revolve around providing information to this cohort, so they are aware of community-based initiatives to support them during public health emergencies, such as those that arise during a pandemic
Silver, H., Rosselot, H., Shaffer, R., & Lozano, R	2022	The impact of the COVID-19 pandemic on school-aged children with Fragile X Syndrome	*Genes*	Survey	Investigate the parental perceived impacts of the pandemic on their children with Fragile X syndrome	33	United States	Parents reported that their children had increased issues with mental health, social skills and sleep. Maintaining face-to-face activities was important	Additional research and investment in the development of resources to support these families are required
Smythe, T., Mabhena, T., Murahwi, S., Kujinga, T., Kuper, H., & Rusakaniko, S	2022	A path toward disability-inclusive health in Zimbabwe Part 2: A qualitative study on the national response to COVID-19	*African Journal of Disability*	In-depth interview	Investigate disabled people’s experiences of accessing health services in Zimbabwe	24	Zimbabwe	The pandemic has had wide-ranging negative impacts on disabled people in Zimbabwe. Access to health and pandemic-related information was interrupted leading to declining physical and mental health. Current policies are inadequate to meet the needs of disabled people, resulting in significant inequities	Because of the severity of the impacts on the health and financial situation of disabled people, there is an immediate need to respond with specific initiatives to alleviate suffering
Solomon Sanders, J., Rajapillai L. I. Pillai, R., Sturley, R., Sillau, S., Asato, M., R., B., Aravamuthan, B., Bonuck, K., Cervenka, M., Hammond, N., Siegel, J., Siasoco, V., & Margolis, B	2022	Impact of the COVID-19 pandemic on the behavioral health of people with intellectual and developmental disabilities	*Psychiatric Services*	Survey	Investigate the behavioural health of people with intellectual disabilities during the pandemic	437	United States	Fifty-two per cent of respondents indicated that their mental health had been negatively impacted because of the pandemic. Access to services to support people were reduced or declined, which correlated with poorer mental health	The pandemic impacted the behaviour of those with intellectual disabilities. Suggestions for making consistent services available for this cohort include using safe social environments and encouraging physical activity to support this cohort's wellbeing
Tetali, S., Kamalakannan, S., Sadanand, S., Lewis, M. G., Varughese, S., Hans, A., & Murthy, G. V. S	2022	Evaluation of the impact of the first wave of COVID-19 and associated lockdown restrictions on persons with disabilities in 14 states of India	*International Journal of Environmental Research and Public Health*	Survey, focus group and interview	Explore the impact of the initial lockdown on disabled people in India	403 survey respondents, 11 interviews	India	Respondents were concerned about infection risk but believed the lockdown negatively impacted their involvement in typical activities. Access to medication and disability services was complicated and led to perceived long-term consequences for health and wellbeing	The pandemic restrictions negatively impact disabled people during the initial lockdown in India. A disability agenda needs to guide and direct the development of policy and strategies to reduce the burdens
Toccalino, D., Haag, H. L., Estrella, M. J., Cowle, S., Fuselli, P., Ellis, M. J., Gargaro, J., & Colantonio, A	2022	Addressing the shadow pandemic: COVID-19 related impacts, barriers, needs, and priorities to health care and support for women survivors of intimate partner violence and brain injury	*Archives of Physical Medicine and Rehabilitation*	Breakout session with semi-structured discussion guide	Investigate the needs, barriers, support services and healthcare requirements for those who have a traumatic brain injury and have experienced intimate partner violence	30	Canada	Intersecting vulnerabilities increased the impact of the pandemic on those with traumatic brain injuries and who have experienced intimate partner violence. Survivors wanted access to formalised peer support and showed an increased need for privacy and projection	Practitioners need education and knowledge of this cohort's health and rehabilitation needs
Turcheti, N., Laurent, A. A., Delgado, C., Sainati, K., Johnson, K., & Wong, E. Y	2022	Social, economic and overall health impacts of COVID-19 on people living with disabilities in King County, WA	*International Journal of Environmental Research and Public Health*	Semi-structured interview	Explore the impact of health, social and economic issues for disabled people because of COVID-19 in the King County, Washington	35	United States	Participants raised issues that impacted the disability community and offered insights regarding how to address their needs during a pandemic	There are lessons that are transferrable to other contexts, such as protocols and processes that have been developed during this study to assist health departments to meet the needs of the disabled community during pandemics. The involvement of the disabled community in planning for health emergency management is key to providing a purpose-built response to this community
Vestal, LE., Schmidt, AM., Dougherty, NL., Sherby, MR., Newland, JG., & Mueller, NB. for the COMPASS-T Study Group	2022	COVID-19-related facilitators and barriers to in-person learning for children with intellectual and development disabilities	*Journal of School Health*	Focus group	Investigate the impact on the education of children with developmental and intellectual disabilities during the pandemic, and to assess the feasibility of weekly testing for this cohort	31 focus groups, 86 participants	United States	COVID-19 testing was difficult for this group but improved a sense of safety when attending in-person schooling	Complying with social distancing is challenging for this population, so frequent testing for COVID-19 is reassuring for families
Waltz, M., Canter, C., Bensen, JT., Berg, JS., Foreman, AK. M., Grant, TL., Hassmiller Lich, K., Navas, A., O’Daniel, JM., Powell, BC., Rini, CM., Staley, BS., & Cadigan, RJ	2022	The burden of COVID-19 on caregivers of children with suspected genetic conditions: A therapeutic odyssey	*Physical & Occupational Therapy in Pediatrics*	Semi-structured interview	Explore the hardship experienced by parents of children who are suspected as having a genetic condition	25	United States	The pandemic did not cause disruptions with the diagnostic process, but people experienced many challenges during this time, including the loss of in-person therapies. Telehealth was problematic leading to concerns about child wellbeing and long-term health	The journey to diagnosis is challenging for these families because the children still need to receive treatment and therapies during a pandemic. Health services need to take into account when planning for future pandemics that this cohort has specific vulnerabilities
Wanjagua, R., Hepburn, S.-J., Faragher, R., John, S. T., Gayathri, K., Gitonga, M., Meshy, C. F., Miranda, L., & Sindano, D	2022	Key learnings from COVID-19 to sustain quality of life for families of individuals with IDD	*Journal of Policy and Practice in Intellectual Disabilities*	Literature review and autoethnographic	Explore the experience of the pandemic on those families with developmental and intellectual disabilities	5	Multi-national	The pandemic had impacts on myriad domains, including interrupting children's education, limiting access to disabilities services and healthcare, negatively impacting family wellbeing	Initiatives such as telehealth helped support families during the pandemic, but the loss of health services, lack of prioritisation for vaccination programmes and loss of services impacted families and resulted in significant disruption and stress. Lessons learned from the pandemic can inform future emergency planning that is inclusive of this cohort
Xu, D., Ma, S., Yan, C., & Zhao, Z	2023	Technology challenges among deaf and hard of hearing elders in China during COVID-19 pandemic emergency isolation: A qualitative study	*Frontiers in Public Health*	In-depth interview	Investigate the technology challenges during the pandemic for those who are Deaf or have hearing impairments	13	China	Participants had issues using technology to enable them to access healthcare; this resulted in an inability to access healthcare, social isolation and lack of engagement with technology	Emergency management systems need accessible policy and equipment to respond to this populations’ needs during pandemics
Zebehazy, KT., Rosenblum, LP., & Thompson, KM	2022	The impact of COVID-19 on transportation of adults with visual impairments	*Journal of Visual Impairment & Blindness*	Open-ended survey	Explore the impact on transport for those with vision impairments during the pandemic	1162	Canada	Vision-impaired people experienced a range of transportation challenges during the pandemic, including when accessing medical care. These challenges were faced financially, within disability support and other networks, and in concerns around family members supporting the vision-impaired person	This research demonstrated that those with visual impairments suffered because of system issues that were exacerbated during the pandemic. Specific transport plans need to be developed and implemented for this population group during health crises or emergency situations

**Fig. 2:**
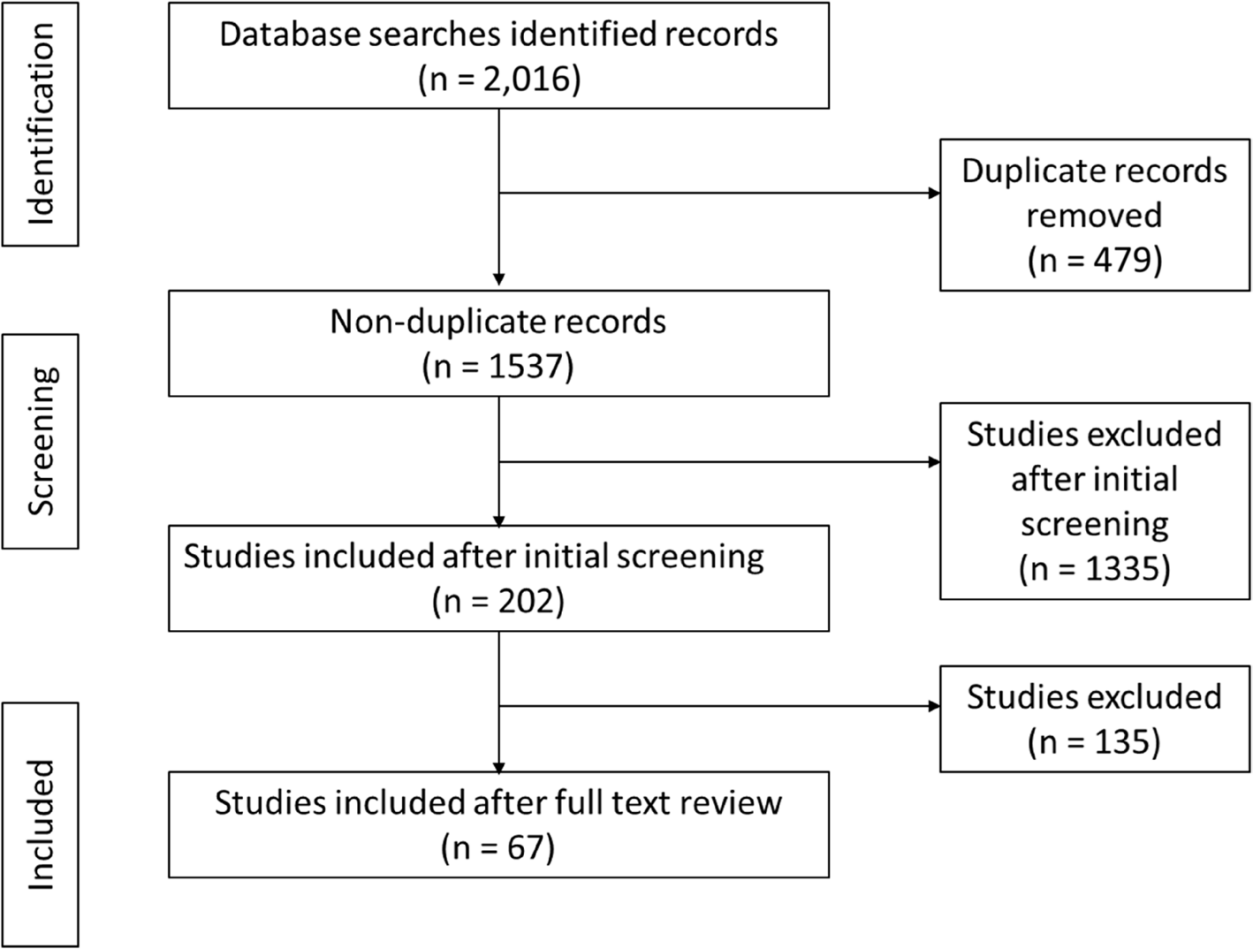
Phase two scoping review literature selection

In phase two, 2016 articles were identified across the various databases: 382 from CINAHL; 42 from OVID; 622 from PubMed; and 970 from Web of Science. Total unique articles once duplicates were removed was 1537; 1431 were published in 2022, and 106 in 2023. Following review of all articles, 1335 were excluded based on assessment against inclusion and exclusion criteria and 20 were screened (Fig. 2). Following screening, 67 articles were in scope (Table 4).

### High-level findings

Amongst the included studies, 59 indicated that the authors employed solely qualitative design [8, 13, 38, 41, 47–101], and another 22 employed either survey or mixed-method approaches [12, 17, 102–121].

Of the 14 studies included in review phase one, none focused solely on healthcare or healthcare access for disabled people during the pandemic. For instance, 14 addressed disabled people’s pandemic experiences [8, 12, 13, 17, 88–91, 93–95, 116, 117, 122], with healthcare access emerging as a sub-topic participants raised. In phase two, 17 authors explored healthcare access [48, 50, 51, 57, 58, 60, 65, 67, 69, 73, 78, 83, 96, 97, 103, 108, 120], and healthcare access arose as a subtopic raised by participants in 50 studies [38, 41, 47, 49, 52–56, 59, 61–64, 66, 68, 70–72, 74–77, 79–82, 84–87, 98–102, 104–107, 109–115, 118, 119, 121].

In phase one, six studies included only those who were disabled in the participant group and 111 from this list of references. Thank you.[8, 13, 90, 92, 95, 96, 111]. Two included parents of disabled children and discussed their experiences of accessing disability rehabilitation services [91, 112]. Five included various participant groups, including those with chronic illness and those who are disabled [17, 89, 93, 94, 111]. No demographic information was available on the final study’s [89] participants because participants were recruited through social media. Phase two paints a different picture with a growing focus on disabled populations, 44 authors focussed solely on disabled participants [38, 41, 47, 48, 50, 51, 53–55, 57–61, 64, 66–69, 72–76, 78–86, 98, 99, 104, 106, 108–110, 114, 115, 117, 120], of these 34 included the voices of parents/ caregivers as well as, or in place of a disabled person [38, 49, 52, 53, 55, 56, 58, 62, 63, 65, 66, 70–72, 76, 77, 79, 87, 88, 97, 100–103, 105–108, 116, 118–122].

### Themes

Four themes emerged from both scoping review phases: *Disrupted healthcare and disability services*, *Mental distress and health services*, *Healthcare access as a biproduct*, and *Faceless minorities*. These are explored in the following sections.

#### Disrupted healthcare and disability services

The most significant outcome from this scoping review was the magnitude of disruption to healthcare and disability services for disabled people. The pandemic exacerbated pre-existing challenges of accessing healthcare [5, 12, 17, 41, 51–54, 58, 59, 65–68, 70–72, 75–79, 81–85, 87, 88, 91, 95, 101, 102, 104, 109, 111, 114, 115, 121], including through attitudes and actions that negatively impacted the quality of health services disabled people received. Precarious financial positions further complicated disabled people’s healthcare access including to necessary medicines [38, 41, 48, 54, 58, 60, 63, 64, 67, 68, 71, 72, 74, 77, 81, 83–85, 101, 104, 109, 111, 114, 115]. Regardless of physical, sensory, or intellectual disabilities, authors highlighted problematic issues in accessing routine healthcare, COVID-19 testing and vaccination; issues included such things as not being able to afford masks, inaccessible venues, peoples’ use of masks creating communication barriers, or a lack of transport [12, 13, 54, 58, 59, 65, 69, 71, 72, 77–83, 85, 91, 95, 101, 109, 114–116, 121].

In some studies, access to healthcare was via telehealth, which was helpful for those with access to this mode of support [12, 17, 23, 53, 55, 84, 90, 91, 95, 114], but further compounded a lack of access for others [54, 57, 59, 65, 78, 84, 87, 88, 93, 95, 101, 111]. For example, access to telehealth was challenging for those who were Deaf, had dementia or an intellectual disability, or could not access or use the internet [12, 13, 51, 57, 59, 78, 84, 87, 91, 95, 101, 111, 112, 116]. Selick and colleagues endorsed individualised approaches when delivering telehealth for people with intellectual or developmental disabilities, as this enhanced the therapeutic value [57]. In addition, some authors indicated there was reduced efficacy for using telehealth for services such as physical therapy [12, 51, 54, 56, 70, 87, 91, 101, 104, 105, 108, 110, 112, 117, 121].

Public health communication tailored for people in the disability community was often missing or not provided in fully accessible formats [38, 41, 52, 58, 59, 67, 73, 74, 78, 83, 85, 98, 101, 114, 115]. Studies highlighted the value of social media or established community networks to develop messaging for the disability community and support information sharing [66, 68, 70, 71, 89, 98]; Dia and Hu’s research demonstrated that using the community to drive accessibility of messaging resulted in agency within the community and effective communication [89]. Other researchers also noted the value of initiatives such as this to offset the significant hardship caused by a lack of disability and healthcare service access during the pandemic [17, 54, 68, 88, 93, 114].

However, for participants in most studies, limited key information about how to access healthcare exacerbated challenges they faced, because people were unsure whether they were able to, or should, access healthcare [12, 13, 41, 52, 58, 59, 65–69, 71–73, 79, 81, 83, 88, 89, 95, 101, 104, 114–116, 121]. Compounding issues, participants in some studies noted that health services in their countries were not offered as COVID-19 led to a reallocation of health practitioners to other services [58, 71, 72, 81, 101]. Additionally, when accessing healthcare, communication was further hampered by healthcare practitioners wearing masks as people with vision, intellectual and hearing impairments struggled to understand verbal instructions and directions, as noted by many authors [12, 13, 41, 52, 59, 65–67, 73, 78, 83, 85, 91, 95, 101, 115, 116]. Participants also highlighted that they did not understand the information health professionals provided but felt too insecure to ask for clarification on care instructions and medication administration [59, 95, 101].

Other authors raised issues about accessing healthcare without disability service support, which meant disabled people were unable to attend clinics in-person because of issues such as a lack of accessible venues, transport or sign language interpreters [12, 13, 54, 65, 68, 71, 72, 78, 82, 83, 85, 94, 104, 115, 117, 121]. Equally, practitioners lack of understanding about specific needs of people trying to access healthcare further intensified access challenges [59, 78, 84, 85, 101, 115]. For example, if doctors failed to recognise and understand specific safety needs then healthcare was inaccessible because of provider issues [59, 78, 84, 85, 101, 115]. Challenges accessing healthcare led disabled people to describe themselves as invisible or completely alone; this perception of invisibility extended across all facets of society with which they interacted [13, 41, 68, 71, 72, 79, 81, 82, 121].

Moreover, fear tempered individual willingness to access healthcare services [13, 41, 53, 58, 65, 67, 68, 71, 72, 76, 79–81, 85, 87, 88, 90, 91, 101, 104, 114, 115, 121]. These fears stemmed from concerns about catching COVID-19 and the rationing of healthcare services, which led some to not access timely healthcare [13, 53, 54, 66, 71, 72, 76–78, 80, 82, 85, 87, 88, 90, 91, 104, 109, 111, 114, 115, 117], resulting in self-reported poorer health outcomes [13, 54, 58, 65, 67, 68, 71, 72, 78, 82, 85, 87, 91, 104, 109, 111, 114, 117, 121]. In turn, people with limited access to support to attend healthcare appointments and consequent compromised healthcare access also had significant safety concerns about surviving serious illnesses [13, 48, 58, 67, 71, 72, 77–79, 85, 104, 109].

Some disabled people encountered a complete cessation of disability services [8, 13, 41, 54, 68, 70–72, 82, 91, 101, 105, 107, 109, 111, 113, 114]; for example, home-based carer support, transport to therapy or healthcare settings. Some studies highlighted that accessible environments, such as therapy pools and day schools, were closed [12, 52, 54, 75, 82, 87, 91, 102, 113, 117, 121]. For other disabled people, access to services was reduced [12, 13, 17, 41, 51, 53–56, 64, 65, 68–71, 75, 79, 82, 83, 85–88, 90, 91, 102, 104–115, 117, 121]. This access change contributed to concerns from disabled people and their parents or caregivers around how the disabled person would maintain their health [8, 12, 13, 17, 41, 51, 52, 55, 56, 64, 70–72, 75, 76, 78, 82, 83, 85–87, 101, 102, 105–108, 110, 114, 115, 117]. Other work highlighted that families also restricted the movements of disabled people because of health concerns, including concerns around the disabled person contracting COVID-19 [8, 13, 76, 86, 90, 101, 117].

#### Mental distress and health services

Research participants used words such as “fearful”, “shocking”, “anxiety producing”, “overwhelming”, “imprisoned” and “isolating” to describe their pandemic experiences [8, 12, 13, 17, 41, 52–55, 58, 59, 64–72, 74, 75, 77–82, 84, 85, 87, 88, 90, 101, 104, 105, 109–111, 114, 116, 117, 121, 122]. These comments stemmed directly from the lack of disability services, which disabled people relied on for the basics of daily life, such as food and medications. As a result, mental health and associated services were discussed widely [8, 12, 17, 53, 55, 58, 64, 65, 68, 70, 75, 85, 88, 91, 92, 104, 106, 110, 114]. The impact of a loss of services led disabled people and their family-carers to experience diminished wellbeing because of a loss of routines and social isolation [8, 12, 13, 41, 52–55, 58, 59, 64–67, 70–73, 75–82, 84–87, 92, 93, 101, 102, 104–107, 109, 111, 113, 114, 117, 121, 122]. Notably, many of these experiences were directly attributable to a lack of access to disability services during the pandemic.

Researchers emphasised the need to consider the United Nation Convention on the Rights of Disabled People and address wider health determinants when planning healthcare services for disabled people during a pandemic [13, 41, 52, 54, 58, 64–66, 70–72, 74, 77–79, 81, 85, 93, 101, 107, 109, 114]. However, accessing mental health services emerged as highly problematic [8, 13, 17, 55, 75, 84, 85, 88, 93, 104]. Participants highlighted that they experienced despair and severe mental distress because of the pandemic [38, 41, 55, 58, 67, 68, 71, 72, 74, 75, 81, 82, 85, 93, 109, 111]. Significantly, a sense of despair and severe mental distress was reported by the most vulnerable disabled people as they had compounding, or intersecting, difficulties including age, identity, ethnicity and geography [13, 41, 54, 58, 64, 67, 68, 71–74, 77, 79, 81, 82, 84, 85, 88, 93, 101, 109, 114, 115]. Notably, several research participants accessed mental health and intellectual disability support virtually, which was beneficial for them [17, 53, 55, 84, 85, 90, 91, 110, 111, 114]; however, such approaches were not universally accessible, leaving some disabled people with no available options for accessing help and support [41, 71, 84, 85, 88, 93, 104, 106, 111, 114]. For those with Fragile-X Syndrome and autistic people, in-person mental health services were preferred because of relationship challenges that resulted from telehealth [55, 77, 112].

#### Healthcare access as a biproduct

As previously mentioned, half the reviewed studies did not specifically focus on healthcare access and, instead, authors canvassed broader areas such as pandemic experiences. Authors focused on health promotion [88], general pandemic communication [95], COVID-19 vaccinations [83], telehealth [57], impact on disability services [109], social media messaging [89] and the pandemic’s acute mental health impacts [75, 92, 93]. Studies also indicated that loss of disability services during the pandemic contributed to disabled people’s isolation, both physically and emotionally, and resulted in heightened mental distress [8, 12, 13, 38, 41, 48, 52–55, 58, 59, 63, 67–72, 75, 77, 79, 82, 91–93, 101, 102, 105, 106, 113–115, 122]. As part of this wider pandemic experience, authors revealed that disabled people developed coping skills and adapted as best they could to the challenging situations in which they found themselves to mitigate their mental distress [58, 79–81, 109, 115]. Healthcare access often arose as a research biproduct and a core issue that impacted them in either positive or negative ways as part of their pandemic experience. This highlights the wide effect limited healthcare access can have on everyday life.

#### Faceless minorities

Disabled populations who experienced compounding and intersecting vulnerabilities were reported to be at a significantly greater risk of experiencing inaccessible healthcare services and bore a greater burden because of the pandemic [38, 41, 48, 58, 71, 72, 77, 103, 114]. For example, the lack of disability services meant that some participants belonging to ethnic minorities or from low to middle-income countries went without their basic needs, this included a lack of access to food and regular medications [38, 48, 63, 77, 114]. Disabled people comprise a significant proportion of the world’s population [2], and the reviewed work originated from around the world. This review, therefore, underscores the unequal burden disabled people have experienced during the pandemic, particularly if residing in middle- to low-income countries [38, 41, 48, 58, 71–73, 77, 81, 101, 114]. Noting a lack of diversity in the inclusion of disabled people and minority populations, authors within the reviewed studies called for diversity of representation in pandemic research and disability-inclusive emergency planning [13, 17, 38, 41, 48, 58, 71–73, 77, 81, 101, 114].

### Research context sub-analysis

An additional analysis was conducted to explore the community of researchers publishing on disabled peoples’ experiences during the pandemic (Table 3). This analysis focussed on identifying characteristics of the primary author, funding source, and explicit connections with the disability community to inform future research. Sub-analysis results revealed only 17 publications that included someone in the authorship group with clearly identified lived experience of disability or who acted as a family-member advocate [13, 17, 38, 55, 63, 65, 68, 78, 79, 83, 89, 91, 95, 105, 108, 101]. Seven phase one authors acknowledged receiving research funding [13, 88, 91, 92, 95, 116, 117], with one other research group indicating they had received partial funding for their study; in phase two, 41 authors indicated that they received funding [38, 47, 49–52, 55, 57–61, 64–66, 68–75, 78, 79, 82, 83, 85, 87, 97–99, 103, 104, 107, 109, 111, 112, 114, 118, 120].

## Discussion

This scoping review, which identified 81 studies, conducted in two distinct phases, is the first to examine disabled people’s experiences of accessing healthcare services during the COVID-19 pandemic. There were 17 studies specifically focused on health service access that involved disabled people as either survey respondents or qualitative participants [48, 50, 51, 57, 58, 60, 65, 67, 69, 73, 78, 83, 96, 97, 103, 108, 120]. These focused on a diverse range of topics, from vaccination experiences [83] to access to safe water [73] during the pandemic. The present scoping review has the unique advantage of showing how pandemic research has grown to form a strong basis on which to advocate for lived-experience research, particularly given the dearth of research in phase one of this review. Continued research is needed to ensure that lived-experience research informs responsive and accessible healthcare service provision for disabled people, especially in emergencies.

When closely scrutinised, the volume of research initially identified by the two review phases (*n* = 3,174) did not include many studies solely focussing on the voices of disabled people. Studies employing a solely qualitative design (*n* = 59) revealed sobering experiences for disabled people in accessing health or disability services; the cessation of in-person disability services seriously impacted disabled people’s quality of life. Although a full exploration of disabled people’s experiences accessing disability services during the COVID-19 pandemic is beyond the scope of the current review, this area must be examined in more depth.

Tellingly, this scoping review revealed that disabled people’s healthcare access needs are not adequately met, especially for those with compounding vulnerabilities who are reliant on disability services. The pandemic has been highly problematic; for disabled people, routine healthcare services all but ceased and services that continued differed vastly from usual. The challenges experienced and lack of health professional response to disabled people’s needs, even when specifically raised, left disabled people feeling invisible [13, 41, 68, 71–73, 81, 82, 121]. Telehealth mitigated some issues but compounded problems for those with certain disabilities, and those without access to telecommunications devices or the internet. In addition, according to research participants, the loss of access to medication and services such as physical and occupational therapy impacted their current and future health status.

### Healthcare planning

Health service planning during pandemics should include a focus on initiatives to improve the wellbeing of disabled people and their families, either in biological or friendship groups. Such wellbeing planning could help mitigate the emotional load associated with isolation. Most importantly, disabled people’s preferences around receiving mental health services during national emergencies, such as pandemics, should be the focus of further research to inform service planning.

The first-hand experiences of the disabled community must be sought so that healthcare and disability services can orient to, and reflect the needs of disabled people. Experts highlight an urgent need to respond to disabled people’s needs across all health system levels [2, 37], which is in keeping with the United Nations Convention on the Rights of Persons with Disabilities [123]. Hochman and colleagues described how policies are disability-blind, and, therefore, disabled people’s needs are not met when delivering healthcare [105]. Moreover, emergency healthcare responses need to be planned systematically and oriented according to community needs and with disability community input. Research by Xu and colleagues [95] highlights how disabled communities can be mobilised to reach those within their communities, meaning that disabled people are willing to support care initiatives. Additional research on the long-term ramifications of disrupted healthcare access for disabled people is pertinent to inform healthcare management going forward.

Another way of addressing the needs of disabled people might be through mapping scenarios using futurist methods that enable people to identify and understand potential unanticipated outcomes from global emergencies [124]. This approach to planning would promote better healthcare and disability service management during such emergencies. However, disabled people must be involved in scenario planning because their unique insights and experience would ensure that any scenarios and subsequent planning would provide for their healthcare and disability service needs.

### Disability-led research

The authors of this review are a group of researchers, including clinician researchers (GG, KMH, TNO) with personal lived experience of disability and/or caring for disabled family members. We have first-hand experience of difficulties accessing healthcare during the pandemic. Our sub-analysis that assessed if disabled researchers were involved in disability research and the availability of funding supporting research endeavours shines a spotlight on the additional vulnerability of disability research.

Unlike the growing body of COVID-19 research on the general population’s experiences, there is limited research on disabled people’s experiences by disabled people. Only six of the 14 reviewed papers in phase one, and eleven in phase two, indicated that the research authorship team included those with lived experience of disability. It is well recognised that disabled researchers are less likely to receive research funding and have been disproportionately impacted by the pandemic [125, 126], our findings confirmed this with 12 research teams indicating they had research funding and also had disabled researchers in the authorship team [13, 38, 55, 65, 68, 78, 79, 83, 91, 95, 98, 127]. Two of these studies come from this very team. Without the inclusion of disabled people, or family advocates, in health services research teams we suspect that healthcare research within the disability field will remain a research-by-product and not provide cogent recommendations for how to change health service delivery.

We contend that disabled researchers, particularly those conducting health research, must be supported by their institutions to carry out timely research that reflects and supports their community. Strategic funding should be made available to help this important cohort in our academic and clinical settings; a call supported by authors whose research was included in this review [77]. The disabled community should be privileged when assigning research funding on disability issues. Editors should also require information on the inclusion of disabled researchers in research that addresses disabled community issues.

### Limitations

To our knowledge, this is the first scoping review that aims to summarise the current understanding of disabled people’s access to healthcare and disability services during the COVID-19 pandemic. Key databases were searched and relevant search terms used to collect as much literature as possible. Challenges in identifying published research could relate to studies being excluded because they (1) do not describe specific conditions as disabilities or use disability-specific keywords or subject areas, or (2) are not published in English. The former challenge highlights the importance of standardised search terms for disability and healthcare research; to mitigate the former challenge we deliberately chose to run a broad search strategy.

## Conclusion

The COVID-19 pandemic experience has generated significant amounts of research, but only a small segment of this has focused on disabled people’s healthcare experience explicitly. There are many valuable lessons to be learnt from such research that can inform solutions for those accessing healthcare. These lessons become increasingly important because of the rise in disability due to long-COVID and an ageing global population. Enhanced health service planning to support disabled people during pandemics is best achieved by including disabled people in pre-pandemic, pandemic, and post-pandemic health system planning. Furthermore, this scoping review demonstrates an urgent need to fund research and charge health systems to be more responsive and inclusive to those in our community who are disabled. This means ensuring strategic support for disabled researcher development, capability building, and research investment.

## Data Availability

All data generated or analysed during this study are included in this published article [and its supplementary information files].
